# Associations between Ionomic Profile and Metabolic Abnormalities in Human Population

**DOI:** 10.1371/journal.pone.0038845

**Published:** 2012-06-13

**Authors:** Liang Sun, Yu Yu, Tao Huang, Peng An, Danxia Yu, Zhijie Yu, Huaixing Li, Hongguang Sheng, Lu Cai, Jun Xue, Miao Jing, Yixue Li, Xu Lin, Fudi Wang

**Affiliations:** 1 Key Laboratory of Nutrition and Metabolism, Institute for Nutritional Sciences, Shanghai Institutes for Biological Sciences, Chinese Academy of Sciences and Graduate School of the Chinese Academy of Sciences, Shanghai, China; 2 Key Laboratory of Systems Biology, Shanghai Institutes for Biological Sciences, Chinese Academy of Sciences and Graduate School of the Chinese Academy of Sciences, Shanghai, China; 3 Department of Endocrinology, Shanghai Xuhui District Central Hospital, Shanghai, China; 4 Departments of Pediatrics, University of Louisville, Louisville, Kentucky, United States of America; 5 Department of Hematology, The First Nanjing People Hospital, Nanjing Medical University, Nanjing, China; 6 Life Science and Chemical Analysis Group in Agilent Technologies Company Limited, Shanghai, China; 7 Shanghai Center for Bioinformation Technology, Shanghai, China; Brigham & Women’s Hospital, and Harvard Medical School, United States of America

## Abstract

**Background:**

Few studies assessed effects of individual and multiple ions simultaneously on metabolic outcomes, due to methodological limitation.

**Methodology/Principal Findings:**

By combining advanced ionomics and mutual information, a quantifying measurement for mutual dependence between two random variables, we investigated associations of ion modules/networks with overweight/obesity, metabolic syndrome (MetS) and type 2 diabetes (T2DM) in 976 middle-aged Chinese men and women. Fasting plasma ions were measured by inductively coupled plasma mass spectroscopy. Significant ion modules were selected by mutual information to construct disease related ion networks. Plasma copper and phosphorus always ranked the first two among three specific ion networks associated with overweight/obesity, MetS and T2DM. Comparing the ranking of ion individually and in networks, three patterns were observed (1) “Individual ion,” such as potassium and chrome, which tends to work alone; (2) “Module ion,” such as iron in T2DM, which tends to act in modules/network; and (3) “Module-individual ion,” such as copper in overweight/obesity, which seems to work equivalently in either way.

**Conclusions:**

In conclusion, by using the novel approach of the ionomics strategy and the information theory, we observed potential associations of ions individually or as modules/networks with metabolic disorders. Certainly, these findings need to be confirmed in future biological studies.

## Introduction

Emerging evidence has suggested that ion homeostasis may play important roles in the global epidemic trend of obesity and related metabolic abnormalities, such as insulin resistance, metabolic syndrome, and type 2 diabetes. Higher body iron (Fe) stores were reported to predict hyperglycemia and type 2 diabetes by some prospective studies [1], [2], [3], whereas higher serum magnesium (Mg) levels were associated with lower risks of metabolic syndrome and type 2 diabetes [4], [5]. Moreover, higher dietary intakes of calcium (Ca), Mg and zinc (Zn) were also related with lower incidence of type 2 diabetes [6], [7], [8], [9], [10]. Meanwhile, data from intervention study also demonstrated that Ca and Zn supplementation could significantly improve fasting glucose levels and insulin resistance [11], [12]. However, most of the previous studies have focused on the role(s) of single or a few ions simultaneously. Given delicate homeostatic controlled nature, relationships of multi-ions are extremely complicated with synergistic or antagonistic interactions under various physical and pathologic conditions [13], [14]. Owing to methodological limitation which could only estimate a few ions at one time, it remains unclear whether a single ion alone or a network of several ions affects metabolic outcomes in a different manner. With recently advanced ionomic technology combining with a mutual information approach which studies multiple ions and their interactions globally as ion modules and/or ion networks, we therefore are able to elucidate complicate associations of ions with metabolic abnormalities systematically.

In recent years, “omics” strategy in the combination with complex multivariate statistical analysis has been extensively applied to discriminate organic bimolecular and reveal biomarkers or patterns in sampled subpopulations in a comparative quantification manner [15]. Introduced by Lahner and colleagues, the term of ionome means the inclusion of all metals, metalloids, and nonmetals presented in an organism [16], and has been extended as metallome that includes biologically significant nonmetals such as phosphorus (P), sulfur (S), selenium (Se) [17], [18]. However, little is known regarding to the information of the ionome so far [19], [20]. Because ionome involves such a broad range of important biological processes, including electrophysiology (potassium [K], sodium [Na]), signal transduction (Ca, Zn), enzymology (copper [Cu], Zn, Se) and structural integrity (Zn, Fe), understanding of the role(s) of ionomic profile and its association with metabolic abnormality will provide novel mechanistic insights linking ion homeostasis and metabolic consequences.

One of major challenges related with analysis of complicated ion network is that traditional methods like principal component analysis (PCA) which is usually based on linear dependences to construct patterns for data with high dimension [21]. However, the interaction among multiple ions might result in much more complex relationships within ion modules/networks than simply linear dependences [13], [14]. To overcome this problem, we applied mutual information in our analysis. Mutual information is a measurement used to quantify the mutual dependence between two random variables [22], [23], and can be applied for either linear or non-linear dependence [24], [25]. This method has been widely used in measuring the co-expression of genes for microarray data analysis [26], and in applying machine learning approaches of bioinformatics, such as feature selection [27]. It was indicated by number of studies that mutual information was more accurate than analysis of variance (ANOVA) and Kruskal-Wallis test in detecting associations [28]. Therefore, by combining the advanced ionomic with mutual information approach, we systematically investigated the ionomic profile, represented by ion modules and/or networks, and their associations with overweight/obesity, metabolic syndrome and type 2 diabetes in Chinese men and women.

## Methods

### Ethics Statement

The study was approved by the Institutional Review Board of the Institute for Nutritional Sciences and written informed consent was obtained from each participants.

### Study Population and Sample Collection

This study used a population-based case-control sample including 559 overweight/obese (body mass index [BMI]≥24.0 kg/m^2^) and 500 age and sex matched normal-weight (18≤BMI<24.0 kg/m^2^) individuals aged 35 to 54 years living in Shanghai, China. The detail of the study was described in elsewhere [29]. Home interviews were conducted by trained health professionals and information on demographics, health status and behaviors were collected through standardized questionnaires [29]. All participants were subsequently invited to take a physical examination after overnight fasting in the local Centers for Disease Control and Prevention (CDC) and community clinics. Body weight, height, waist circumference, and blood pressure were measured by a standardized protocol [29]. Individuals without data of ion (n = 83, 7.8%) were excluded and a total of 976 participants was included in the final analyses.

During the physical examination, fasting peripheral venous blood was collected by a tube containing anticoagulant and then centrifuged at 4°C, 3000 rpm for 15 min. All samples were shipped in dry ice to the Institute for Nutritional Sciences and stored at −80°C until analysis. Measurements of total cholesterol, high-density lipoprotein (HDL) cholesterol, low-density lipoprotein (LDL) cholesterol, triglycerides, glucose, C-reactive protein (CRP), and interleukin-6 (IL-6) were described previously [29].

Metabolic syndrome was defined according to the updated National Cholesterol Education Program-Adult Treatment Panel III criteria for Asian-Americans [30], including at least 3 of the following components: 1) Waist circumferences ≥90 cm in men or ≥80 cm in women; 2) Triglycerides ≥1.7 mmol/l; 3) HDL cholesterol <1.03 mmol/l in men or <1.30 mmol/l in women; 4) Blood pressure ≥130/85 mmHg, or current use of anti-hypertensive medications; and 5) Fasting plasma glucose (FPG) ≥5.6 mmol/l. Type 2 diabetes was defined as FPG ≥7.0 mmol/L or 2-h postload plasma glucose ≥11.1 mmol/l during an oral glucose tolerance test (OGTT). OGTT was conducted in 476 subjects with 5.5≤FPG<7.0 mmol/l during the physical examination. The selection procedure for OGTT was described elsewhere [29].

### Laboratory Measurements for ICP-MS Detection

#### Instrument and reagents

Agilent 7500cx inductively coupled plasma mass spectroscopy (ICP-MS) system (Agilent Technologies, Tokyo, Japan) equipped with a G3160B I-AS integrated autosampler was employed to measure ion profile. G3148B ISIS system was also used to reduce detection time and volume of each sample. Ni sample cone and skimmer cone were utilized with an orifice diameter of 1.0 and 0.4 mm, respectively. Sample introduction was performed with a micromist nebulizer combined with a Scott-type double pass spray chamber (Agilent Technologies). The instrument was tuned to optimal conditions in terms of sensitivity (Li, Y, Co, and Tl) and CeO/Ce and Ce^2+^/Ce by using a tuning solution (Agilent Technologies) containing 1 µg/L of Li, Y, Tl, Ce and Co in 2% HNO_3_ (w/v). The determination was operated in full quantitative mode, and typical operating conditions used in this study were summarized in **Table S1**.

**Table 1:** Characteristics of participants according to obese status (n = 976).

	Normal-weight	Overweight/Obesity	
	18≤BMI<24	BMI≥24	P value
N	460	516	
Age (yrs)*	45.8 (5.5)	46.0 (5.3)	0.487
Men (n, %)*	149 (32.4)	207 (40.1)	0.012
Physical inactivity (n, %)	230 (50.0)	256 (49.6)	0.912
Education levels (n, %)			0.004
0∼9 yrs	105 (22.8)	159 (30.8)	
10∼12 yrs	247 (53.7)	260 (50.4)	
>12 yrs	108 (23.5)	97 (18.8)	
Current smoker (yes, n, %)	97 (21.1)	137 (26.6)	0.687
Alcohol drinker (yes, n, %)	168 (36.5)	183 (35.5)	0.106
Family history of chronic diseases (n, %)	186 (40.4)	209 (40.5)	0.972
Metabolic syndrome (n, %)	43 (9.4)	356 (69.0)	<0.001
Type 2 diabetes (n, %)	26 (5.7)	96 (18.6)	<0.001
BMI (kg/m^2^)	21.0 (1.4)	27.9 (2.6)	<0.001
Waist circumference (cm)	75.7 (6.1)	92.9 (7.8)	<0.001
Systolic blood pressure (mmHg)	118.0 (15.1)	130.5 (17.5)	<0.001
Diastolic blood pressure (mmHg)	74.4 (9.7)	83.7 (11.4)	<0.001
Glucose (mmol/l)	5.76 (1.05)	6.26 (1.49)	<0.001
Insulin (µU/ml)†	7.4 (7.0–7.7)	11.2 (10.8–11.7)	<0.001
HOMA-IR†	0.85 (0.82–0.89)	1.31 (1.26–1.37)	<0.001
Total cholesterol (mmol/l)	5.14 (1.15)	5.30 (1.15)	0.020
LDL cholesterol (mmol/l)	3.12 (0.94)	3.40 (0.96)	<0.001
HDL cholesterol (mmol/l)	1.53 (0.44)	1.24 (0.34)	<0.001
Triglycerides (mmol/l)†	0.99 (0.94–1.04)	1.57 (1.49–1.65)	<0.001
CRP (mg/l)†	0.60 (0.55–0.65)	1.35 (1.24–1.46)	<0.001
IL-6 (pg/ml)†	1.19 (1.12–1.26)	1.66 (1.57–1.75)	<0.001

*P* value was calculated after adjustment for age and sex. Data are arithmetic mean (SD). Percentages may not sum to 100 because of rounding.

* Data not adjusted for itself.

† Data are geometric mean (95% CI).

The internal standard containing Ge, ^6^Li, Lu, Rh, Tb, Sc, In, and Bi (SPEX CertiPrep) was injected by peristaltic pump into the ion source at an approximate concentration of 500 µg/L in the online mode. The multi-element standard solution containing aluminum (Al), arsenic (As), boron (B), barium (Ba), beryllium (Be), bismuth (Bi), Ca, cadmium (Cd), cobalt (Co), chromium (Cr), Cu, Fe, K, lithium (Li), Mg, manganese (Mn), molybdenum (Mo), Na, nickel (Ni), P, lead (Pb), palladium (Pd), rhenium (Re), S, antimony (Sb), Se, silicon (Si), stannum (Sn), strontium (Sr), vanadium (V), titanium (Ti), wolfram (W) and Zn, each at a concentration of 1000 µg/mL (SPEX CertiPrep) were used. We detected 17 elements of them including Cr, Mn, Fe, Zn, Cu, Se, Mo, Sr, Sn, Sb, Ti, Mg, Ca, P S, K, and Re. The standard curve included 0, 0.1, 1, 10, 100, 1000 and 10000 µg/L, respectively. Ultrapure water (18.2 MΩ) was obtained from a water-purification system (Sartorius, Arium 61316). Ultrapure grade HNO3 (100ppt, 65% v/v, TAMA) was used in this study.

Each 100 µL of the plasma sample was placed into a 15 mL centrifuge tube coated with PFA, to which 400 µL HNO_3_ was added. The centrifuge tube was then placed into 150°C water bath for 3 h until the solution became clear. The resulting solution was diluted with ultrapure water to about 2 mL. The diluted samples were stored at 4°C for ICP-MS analysis.

#### ICP-MS analytical validation

To test the validity of the whole detection system, we determined concentrations of Na, Mg, K, Ca, Fe, Cu, Zn, and Se in human serum (National Institute of Quality Standards, Beijing, China, GBW09152) and compared them with reference concentrations. Serum samples were pre-treated and determined in the same procedure as described above (**Table S2**). Moreover, the recovery test of 17 elements in human plasma samples was repeated three times. At first, 17 elements were quantified at their original concentrations. Then, the 20 µg/L multi-element standard solution was added to random plasma samples, and the spiked samples were assayed. Recoveries were estimated using the formula: recovery (%)  =  ((amount found- original amount)/(amount spiked)) × 100% (**Table S3**). Additionally, the intra- and inter-day stability of the instrument was assessed by analyzing concentrations of 17 elements in the quality control sample of fetal bovine serum (Sijiqing Co. Ltd., Hangzhou, China) added with 20 µg/L multi-element standard solution. We detected quality control samples after each 10 plasma samples. Moreover, concentrations of 17 elements in one human plasma sample were detected 10 times in parallel to confirm the precision of the method (**Table S4**).

**Table 2:** Characteristics of ion concentrations in study participants.

	Overweight/obesity		Metabolic syndrome		Type 2 diabetes	
	No (n = 460)	Yes (n = 516)	No (n = 577)	Yes (n = 399)	No (n = 854)	Yes (n = 122)
Age (yrs)*	45.8 (5.5)	46.0 (5.3)	45.4 (5.5)	46.7 (5.1)‡	45.9 (5.4)	46.2 (5.3)
Men (n, %)*	149 (32.4)	207 (40.1)‡	188 (32.6)	168 (42.1)‡	304 (35.6)	52 (42.6)
BMI (kg/m^2^)	21.0 (1.4)	27.9 (2.6)‡	22.6 (3.2)	27.6 (3.2)‡	24.2 (3.9)	27.2 (4.0)‡
Ca (ppm)†	6.46 (6.39–6.54)	6.54 (6.46–6.62)	6.50 (6.44–6.58)	6.49 (6.40–6.59)	6.51 (6.45–6.57)	6.47 (6.33–6.62)
Cr (ppb)†	0.70 (0.49–0.99)	0.75 (0.54–1.04)	0.52 (0.38–0.72)	1.15 (0.81–1.64)‡	0.66 (0.51–0.85)	1.42 (0.77–2.60)‡
Cu (ppb)†	629.7 (613.7–646.1)	657.3 (640.6–674.5)‡	630.7 (616.2–645.6)	664.0 (644.9–683.7)‡	639.4 (627.2–651.9)	678.0 (641.8–716.4)‡
Fe (ppb)†	818.5 (779.8–859.2)	910.2 (870.0–952.3)‡	847.2 (810.9–885.1)	893.4 (849.2–939.8)	859.6 (829.2–891.2)	909.9 (837.9–988.1)
K (ppm)†	413.0 (403.0–423.2)	410.6 (401.4–420.0)	412.2 (403.3–421.3)	411.0 (400.6–421.6)	411.1 (403.8–418.5)	416.0 (398.0–434.8)
Mg (ppm)†	18.7 (18.5–18.8)	18.4 (18.3–18.6)‡	18.6 (18.5–18.8)	18.4 (18.2–18.6)‡	18.6 (18.5–18.8)	18.0 (17.6–18.4)‡
Mn (ppb)†	1.24 (1.04–1.49)	1.42 (1.23–1.64)	1.24 (1.06–1.44)	1.49 (1.26–1.76)	1.31 (1.16–1.48)	1.52 (1.15–2.01)
Mo (ppb)†	0.76 (0.62–0.94)	0.68 (0.55–0.82)	0.76 (0.63–0.90)	0.66 (0.52–0.84)	0.71 (0.61–0.83)	0.75 (0.51–1.09)
P (ppm)†	75.6 (74.4–76.7)	77.7 (76.6–78.8)‡	74.9 (74.0–75.9)	79.3 (77.9–80.7)‡	76.3 (75.5–77.2)	79.3 (76.8–81.8)‡
Re (ppb)†	0.07 (0.07–0.08)	0.08 (0.07–0.08)	0.07 (0.07–0.08)	0.07 (0.07–0.08)	0.08 (0.07–0.08)	0.07 (0.06–0.07)‡
S (ppm)†	1012.2 (1003.4–1021.1)	1029.1 (1019.7–1038.5)‡	1015.2 (1007.3–1023.1)	1029.7 (1018.7–1040.8)‡	1018.5 (1011.7–1025.4)	1039.4 (1020.2–1059.1)‡
Sb (ppb)†	0.50 (0.40–0.62)	0.38 (0.30–0.48)	0.40 (0.32–0.49)	0.49 (0.38–0.62)	0.42 (0.35–0.50)	0.55 (0.37–0.82)
Se (ppb)†	95.3 (93.7–96.9)	96.8 (95.1–98.4)	94.9 (93.4–96.4)	97.8 (95.9–99.7)‡	95.6 (94.4–96.8)	99.5 (95.6–103.5)‡
Sn (ppb)†	0.91 (0.87–0.95)	0.93 (0.89–0.98)	0.90 (0.87–0.94)	0.95 (0.91–1.00)	0.93 (0.90–0.96)	0.89 (0.82–0.97)
Sr (ppb)†	32.3 (31.5–33.2)	34.0 (33.1–34.8)‡	32.5 (31.7–33.2)	34.2 (33.2–35.3)‡	33.2 (32.5–33.8)	33.2 (31.7–34.7)
Ti (ppb)†	139.8 (138.6–141.1)	140.4 (139.1–141.8)	139.8 (138.7–140.9)	140.6 (139.1–142.2)	139.9 (139.0–140.9)	141.5 (139.1–144.1)
Zn (ppb)†	389.6 (379.2–400.4)	404.7 (394.5–415.2)	393.8 (384.8–403.1)	402.9 (390.7–415.5)	397.5 (389.4–405.6)	398.1 (380.9–415.9)

Data are arithmetic mean (SD).

* Data not adjusted for itself.

† Data are geometric mean (95% CI).

‡ *P* value<0.05 between 2 groups after adjustment for age and sex. Percentages may not sum to 100 because of rounding.

**Table 3:** Partial spearman correlation coefficients between ions and metabolic features and inflammatory markers (n = 976).

	Ca	Cr	Cu	Fe	K	Mg	Mn	Mo	P	Re	S	Sb	Se	Sn	Sr	Ti	Zn
Age*	−0.06	0.15†	0.14†	0.08§	−0.02	0.07§	0.05	0.19†	0.25†	−0.11†	0.03	0.18†	0.15†	0.11†	0.02	0.04	−0.13†
BMI	0.07	0.00	0.13†	0.08§	0.01	−0.08§	0.02	−0.05	0.13†	0.04	0.11†	−0.02	0.04	0.02	0.10‡	0.05	0.09‡
Waist circumference	0.06	0.06	0.17†	0.06	0.01	−0.07§	0.02	−0.04	0.15†	−0.00	0.12†	0.03	0.07§	0.06	0.14†	0.06	0.04
SBP	0.12†	0.01	0.06	0.05	−0.00	−0.03	0.10‡	−0.02	0.17†	0.10‡	0.20†	−0.03	0.06§	0.06	0.10‡	0.14†	0.14†
DBP	0.11†	0.07§	0.10‡	0.05	0.01	−0.01	0.13†	−0.00	0.19†	0.08§	0.19†	0.01	0.08§	0.09‡	0.12†	0.15†	0.14†
Glucose	−0.18†	0.16†	0.13†	−0.04	−0.00	−0.15†	−0.01	0.00	0.02	−0.25†	−0.04	0.13†	0.03	−0.04	−0.02	−0.12†	−0.19†
Insulin	0.01	0.10‡	0.16†	−0.01	0.04	−0.06§	−0.08§	0.07§	0.20†	−0.05	0.15†	0.09‡	0.15†	0.05	0.02	0.04	−0.03
HOMA-IR	−0.00	0.11†	0.17†	−0.01	0.04	−0.07§	−0.08§	0.07§	0.21†	−0.06	0.15†	0.10‡	0.16†	0.05	0.02	0.03	−0.04
Total cholesterol	−0.13†	0.08‡	0.14†	0.03	0.01	−0.03	−0.00	0.00	0.37†	−0.19†	−0.01	0.07§	0.17†	−0.02	−0.02	−0.07§	−0.10‡
LDL cholesterol	−0.08‡	0.07§	0.16†	0.04	0.01	−0.01	−0.00	−0.01	0.32†	−0.13†	0.02	0.06	0.17†	−0.00	0.00	−0.04	−0.05
HDL cholesterol	−0.16†	0.01	−0.06	0.00	−0.01	−0.03	0.02	0.01	0.03	−0.13†	−0.15†	0.04	0.00	−0.05	−0.08§	−0.12†	−0.16†
Triglycerides	−0.02	0.14†	0.19†	0.01	−0.03	−0.02	0.01	0.05	0.40†	−0.13†	0.12†	0.10‡	0.13†	0.05	0.09‡	0.04	−0.01
CRP	0.04	0.07§	0.42†	−0.08§	−0.04	−0.05	0.04	−0.01	0.16†	−0.02	0.06	0.04	0.07§	0.08‡	0.11†	0.05	−0.05
IL-6	0.07§	0.05	0.31†	−0.06	−0.06	−0.00	0.01	−0.03	0.10‡	−0.01	0.07§	0.01	0.00	0.06	0.13†	0.08§	−0.01

All correlation coefficients were calculated after adjustment for age and sex.

* Data not adjusted for itself.

† *P*<0.001,

‡ *P*<0.01,

§ *P*<0.05.

**Figure 1:**
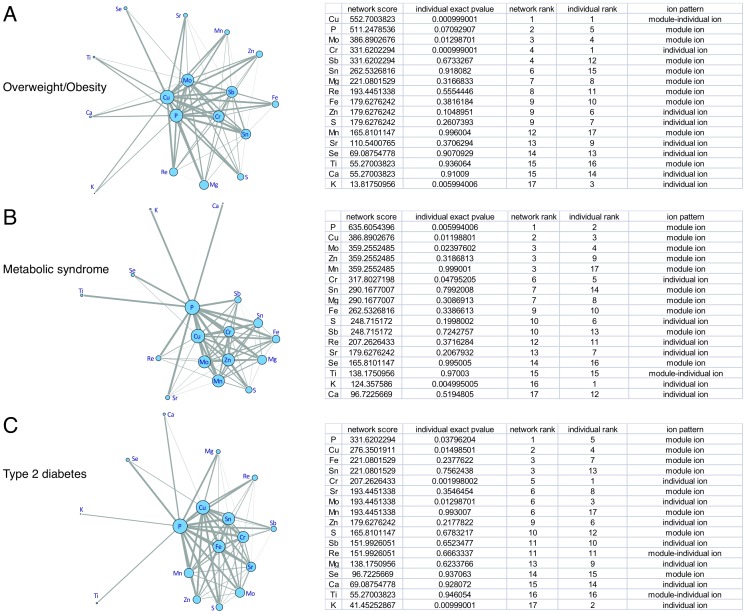
The overweight/obesity, metabolic syndrome and type 2 diabetes related ion network. A. The overweight/obesity related ion network; **B.** The metabolic syndrome related ion network; **C.** The type 2 diabetes related ion network. The size of node represents the combined Fisher score of significant combinations involve a specific ion, which indicates the strength of ion module in association with metabolic disorders. The width of the edge represents the Fisher score of edge between connected ions, which indicates the possibility of forming an ion module associated with metabolic disorders. Three ion patterns were postulated when comparing the rank of ion effect in individual and in network associated with metabolic disorders. “Individual ion” was defined as the rank of ion in the network posterior to that of single ion. “Module ion” was defined as the rank of ion in network prior to that of single ion. “Module-individual ion” was defined as the rank of ion in network equivalent to that of single ion.

### Bioinformatics Analysis

Log-transformations were performed for all 17 ions to approximate normality. Analysis of covariance for continuous variables and logistic regression models for categorical variables were applied for the comparison across different metabolic outcomes.

All ion modules were tested to determine whether they were associated with overweight/obesity, metabolic syndrome or type 2 diabetes based upon information theory. When computing the conditional mutual information between ion module and disease status, the following variables: for overweight/obesity, sex and age were adjusted; for metabolic syndrome and type 2 diabetes, sex, age and BMI were adjusted. Then the significant ion modules were selected to construct relevant ion networks as following three steps:

#### Step 1: Scoring ion modules

An ion module was defined as an ion set. We exhausted all possible combinations with number of ions from 1 to 8. There were 
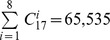
 such ion modules. Given a particular ion module M, let X represent its vector of scores over the samples, and let Y represent the corresponding vector of class labels (disease or normal). To derive a, expression values g_ij_ were normalized to z-transformed scores z_ij_ which for each ion i had mean μ = 0 and standard deviation δ = 1 over all samples j.

The individual z_ij_ of each member ion in the ion module were averaged into a combined z-score, which was designated as x_j_. Many statistical methods, such as the *t* or Wilcoxon score, could be used to score the relationship between X and Y. In this study, we defined the conditional mutual information between X and Y
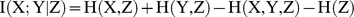
Where X, Y and Z enumerate values of ion module levels, metabolic disorders and adjust variables, respectively. H was the entropy of the empirical probability distribution. To derive a’ from a, activity levels were discredited into [log 2(# of samples) +1] = 11 equally spaced bins [31].

#### Step 2: Searching for significant ion modules

To assess the significance of the identified ion modules, we calculated the exact permutation p-values using the function permp in R package statmod [32]. For the random model, the class labels were permuted 1,000 times, yielding a null distribution of mutual information scores for each ion module; the real score of each ion module was indexed on this null distribution. In this study, the significance of ion modules was selected to satisfy the permutation tests with *P*<0.001, according to the null distributions of S.

#### Step 3: Identifying disease related ion network

After obtained those ion modules that were significantly associated with metabolic abnormalities, we identified three ion networks to illustrate the pattern of ion cooperation. For ion *x* which was in *k* significant ion modules, the combined score using Fisher’s method [33], [34], [35] was
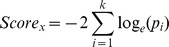
Where *p_i_* was the permutation p-value for ion module *i* which has ion *x*. *Score_x_* had a chi-square distribution with 2*k* degrees of freedom, where *k* was the number of tests being combined.

Within the significant ion module, each two ions were considered as possible edge. So each ion module was a full connected module. All the significant ion modules formed the candidate network. In the network, each edge had a score. For edge between ion x and ion y, the score was
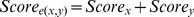



To identify the maximum scored subnetwork, we used a greedy edge expansion algorithm by growing from every locally maximal scored edge whose score was larger than its adjacency [33]. Every growing new edge would get a probability by a hypergeometric distribution:
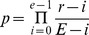



Here *E* was the number of edges in the candidate network and *e* was how many edges had already been included during extension. This value was used to measure the probability to get an edge with higher rank than *r* from the total *E* edges when we obtained the r-rank at present step. The probability equals to 1 when all edges were selected and there was a minimum with the increase of *e*. The minimum one was used as our cutoff to terminate the growth procedure. In this way, the most of significant subnetwork were selected as the identified metabolic outcome related ion network.

To study the module structure of ions, the combined score of ion set was defined as 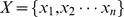 using Fisher’s method [33], [34], [35]

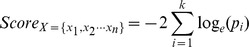
where p_i_ was the permutation p-value for ion module i which included ion set 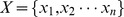 .

## Results

### Characteristics of Ion Concentrations in the Study Population

The study population included 516 overweight/obese and 460 normal-weight participants. The mean BMI values were 27.9±2.6 kg/m^2^ and 21.0±1.4 kg/m^2^ for overweight/obese and normal-weight participants, respectively (*P*<0.001). The prevalence were 40.9% (n = 399) for metabolic syndrome and 12.5% (n = 122) for type 2 diabetes in the study population.

Compared with those of normal-weight subjects, overweight/obese participants were more likely to be male, with lower educational attainment and higher prevalence of metabolic syndrome and type 2 diabetes (all *P*<0.05; **Table 1**). They also exhibited higher values of waist circumference, blood pressure, glucose, insulin, HOMA-IR, total cholesterol, LDL cholesterol, triglycerides, CRP and IL-6, and lower HDL cholesterol concentration (all *P*<0.05).

As shown in **Table 2**, compared to the normal groups, all groups with obesity, metabolic syndrome or type 2 diabetes had elevated levels of Cu, P and S, but lower Mg concentration, after adjusting for age and sex. In addition, participants with overweight/obesity, metabolic syndrome and type 2 diabetes also appeared to have higher levels of Fe and Sr, Cr, Se and Sr, or Cr and Se.

Plasma Cu and P levels were positively associated with most of the metabolic traits listed in **Table 3**. In addition, plasma levels of Ca, Cr, Mg, Re, Sb, Ti and Zn were also significantly correlated with fasting glucose levels while concentrations of Cr, Re, S, Se and Sr were significantly correlated with triglyceride levels. Notably, strong positive correlations were observed between Cu and CRP (*r* = 0.42) and also between Cu and IL-6 (*r* = 0.31) after adjusting for age and sex (all *P*<0.001). Other ions such as P and Sr were also significantly correlated with inflammatory markers (all *P*<0.05).

### Metabolic Disorders Related Ion Modules and Networks

There were 50, 60 and 38 ion modules associated with overweight/obesity, metabolic syndrome and type 2 diabetes with exact permutation *P*-value <0.001, respectively. The number of exact permutation is 65,535 ion modules with overweight/obesity, metabolic syndrome and type 2 diabetes. The complete exact permutation *P* value (<0.01) list of ion modules with overweight/obesity, metabolic syndrome and type 2 diabetes were presented in **Table S5**, **Table S6** and **Table S7**.

The overweight/obesity, type 2 diabetes and metabolic syndrome related ion networks were shown in **Figure 1**. The Fisher score of 68 edges between connected ions in those three networks were also demonstrated in **Table S8**, **Table S9** and **Table S10**. Among the 68 edges of all three networks, Cu-P ranked in the first, suggesting the strongest connected ion pair (**Tables S8, S9,** and **S10**). While Cu-Zn, a previously well studied ion pair, ranked 14^th^ among 68 edges of overweight/obesity network (**Table S8**), 17^th^ among 68 edges of metabolic syndrome network (**Table S9**), and 19^th^ among 68 edges of type 2 diabetes network (**Table S10**).

**Table 4:** Two ion module of Cu and three ion module of Cu and Zn in overweight/obesity.

Two ion module of Cu			Three ion module of Cu and Zn			
Ion 1	Ion 2	Score	Ion 1	Ion 2	Ion 3	Score
Cu	P	428.3428	Cu	Zn	P	124.3576
Cu	Mo	317.8027	Cu	Zn	Mo	82.90506
Cu	Sb	303.9852	Cu	Zn	Sb	82.90506
Cu	Cr	276.3502	Cu	Zn	Mg	69.08755
Cu	Sn	234.8977	Cu	Zn	Cr	27.63502
Cu	Mg	179.6276	Cu	Zn	Fe	27.63502
Cu	Re	179.6276	Cu	Zn	Re	27.63502
Cu	Mn	151.9926	Cu	Zn	S	27.63502
Cu	S	124.3576	Cu	Zn	Se	27.63502
Cu	Zn	124.3576	Cu	Zn	Sn	27.63502
Cu	Sr	110.5401	Cu	Zn	Ca	13.81751
Cu	Fe	96.72257	Cu	Zn	Sr	13.81751
Cu	Ca	41.45253	Cu	Zn	Ti	13.81751
Cu	Se	41.45253				
Cu	Ti	41.45253				

To show the effects of highly connected ion modules, we calculated the combined Fisher score for two ion modules and three ion modules (**Table S11)**. **Table 4**, **Table 5** and **Table 6** showed that two ion modules contained Cu and three ion modules contained Cu-Zn were associated with overweight/obesity, metabolic syndrome and type 2 diabetes (extracted from **Table S11)**. The Cu-P module scored the highest in all two ion modules of Cu. While in three ion modules of Cu-Zn, the Cu-Zn-P module scored the highest for both overweight/obesity and metabolic syndrome, and the Cu-Zn-Mg module scored the highest for type 2 diabetes.

### Postulated Ion Patterns in Association with Metabolic Disorders

**Figure 1** also showed the exact permutation *P*-values of single ion with overweight/obesity, metabolic syndrome and type 2 diabetes, respectively. Plasma levels of Cr, Cu, K and Mo were significantly associated with overweight/obesity; plasma levels of K, P, Cu, Mo and Cr were significantly associated with metabolic syndrome; plasma levels of Cr, K, Mo, Cu and P were significantly associated with type 2 diabetes (all *P*<0.05). Ranks of ions in the networks were also displayed in **Figure 1**. Comparing the rank of ion effect in individual and in network associated with metabolic disorders, three ion patterns were postulated: (1) “Individual ion” was defined as the rank of ion in the network posterior to that of single one, including K and Cr. Those ions were prone to affect metabolic disorders in individual ways; (2) “Module ion” was defined as the rank of ion in network prior to that of single ion, which was prone to affect metabolic disorders in combination with other ions. Fe was not significantly associated with type 2 diabetes, but always occurred in diabetes associated ion modules; and (3) “Module-individual ion”, in that situation ion like Cu ranked equivalently in individual and in network associated with obesity.

## Discussion

To our best knowledge, this was the first study to utilize the novel approach of ionomics with mutual information to investigate simultaneously single ion and multiple ions that were represented by ion modules/networks and the associations with metabolic abnormalities in human population. Three constructed ion networks were found to be associated specifically with obesity, metabolic syndrome and type 2 diabetes. Effects of ion(s) could be postulated as “Individual ion”, “Module ion” and “Module-individual ion”, suggesting potential associations of different ions and/or ion modules/networks with the metabolic risks in the Chinese population.

**Table 5:** Two ion module of Cu and three ion module of Cu and Zn in metabolic syndrome.

Two ion module of Cu			Three ion module of Cu and Zn			
Ion 1	Ion 2	Score	Ion 1	Ion 2	Ion 3	Score
Cu	P	317.8027	Cu	Zn	P	124.3576
Cu	Cr	221.0802	Cu	Zn	Cr	69.08755
Cu	Mo	179.6276	Cu	Zn	Mo	69.08755
Cu	Sn	165.8101	Cu	Zn	S	69.08755
Cu	Sb	151.9926	Cu	Zn	Mg	41.45253
Cu	Zn	138.1751	Cu	Zn	Re	41.45253
Cu	Mg	124.3576	Cu	Zn	Se	41.45253
Cu	Mn	124.3576	Cu	Zn	Sn	41.45253
Cu	Re	124.3576	Cu	Zn	Sr	41.45253
Cu	S	110.5401	Cu	Zn	Ca	27.63502
Cu	Fe	69.08755	Cu	Zn	Fe	27.63502
Cu	Sr	69.08755	Cu	Zn	Sb	27.63502
Cu	Se	55.27004	Cu	Zn	Mn	13.81751
Cu	Ca	41.45253				
Cu	Ti	41.45253				
Cu	K	27.63502				

**Table 6:** Two ion module of Cu and three ion module of Cu and Zn in Type 2 diabetes.

Two ion module of Cu			Three ion module of Cu and Zn			
Ion 1	Ion 2	score	Ion 1	Ion 2	Ion 3	Score
Cu	P	221.0802	Cu	Zn	Mg	55.27004
Cu	Cr	151.9926	Cu	Zn	P	55.27004
Cu	Sn	138.1751	Cu	Zn	Ca	41.45253
Cu	Mo	96.72257	Cu	Zn	Cr	41.45253
Cu	Sr	96.72257	Cu	Zn	S	41.45253
Cu	Mg	82.90506	Cu	Zn	Sb	41.45253
Cu	Mn	82.90506	Cu	Zn	Sn	41.45253
Cu	S	82.90506	Cu	Zn	Mo	27.63502
Cu	Sb	82.90506	Cu	Zn	Fe	13.81751
Cu	Zn	82.90506	Cu	Zn	Mn	13.81751
Cu	Fe	69.08755	Cu	Zn	Re	13.81751
Cu	Re	69.08755	Cu	Zn	Se	13.81751
Cu	Ca	41.45253	Cu	Zn	Sr	13.81751
Cu	Se	41.45253				
Cu	K	27.63502				
Cu	Ti	13.81751				

In this study, using ionomic approach, instead of individual ion, we revealed not only the complicated relationships of ion-ion, ion-phenotypes including obesity, metabolic syndrome and type 2 diabetes, but also specific ion modules/networks, which linked with different stages in obesity related metabolic disorders. Previously, individual role of specific ions like Fe and Zn in the pathogenesis of metabolic disorders have been broadly investigated in cross-sectional studies, prospective studies, and even clinical trials [1], [2], [7], [11], [36]. More recently, some of investigators have used Mass Spectrometry and Atomic Absorption Spectroscopy to measure multi-ions. They also compared individual ion concentrations between obese or diabetic cases and non-obese or non-diabetic controls [37], [38], [39], [40], [41], as well as between diabetic patients with and without metabolic alterations [38]. However, it still remains unknown regarding the cluster effect of ion modules/networks on obesity and related metabolic disorders. Indeed, it was noticed previously that multiple ions may interact synergistically or antagonistically and consequently influence overall ion homeostasis [13], [14]. Moreover, a specific ion when presented alone or in a combination with other ion(s) may have different impact on metabolic outcomes. Therefore, although the ionomic approach provides a great opportunity to study the global effect of multi-ions, it also becomes a big challenge to elucidate complicated relationships and mechanistic linking between multiple ions and metabolic disorders.

To better explore the complex associations between ionomic profile and different stages of metabolic abnormalities in population level, we employed a novel machine learning data processing approach, namely mutual information in the present study. In many prior studies, paired Student’s *t* test, Mann–Whitney U-test or ANOVA have been frequently used to compare ion concentrations in different groups [37], [38], [39], [40], [41], [42]. One study also adopted PCA analysis to explore relationships of metals/metalloids with clinical parameters [42]. Although PCA analysis was admittedly useful to construct patterns from high dimension data, the linear dependence nature of this approach might lose information [21] and could not describe adequately for the non-linear dependences resulting from synergistic or antagonistic interactions within ion modules/networks [13], [14]. On the other hand, the mutual information that measures the general dependence including both linear and non-linear dependences [24], [25], may allow us to discover specific ionomic fingerprints attributing to different metabolic outcomes. Indeed, with this approach, three specific ion networks were constructed to reflect stages of obesity, metabolic syndrome and type 2 diabetes. Certainly, more prospective studies are merited to confirm our findings.

Another noteworthy finding of this study is that we characterized ion modules/networks as “Individual ion”, “Module ion” and “Module-individual ion”, implicating that the role of a specific ion in the pathogenesis of metabolic disorders may vary according to whether single ion or ions in modules/networks were included in the data analysis. For instance, Fe *per se* was not significantly associated with type 2 diabetes, while it ranked 3^rd^ in the ion networks associated with type 2 diabetes. Previously, we have observed that high Fe load, indicated by elevated circulating ferritin, was significantly associated with type 2 diabetes in Chinese population [43]. Prospective studies conducted in Western populations also demonstrated a predictive role of ferritin level in the onset of type 2 diabetes [1], [2]. The discrepancy between the current study and earlier studies might be due to the fact that Fe was used as an ion form rather than its protein biomarker like ferritin. However, given the nature of complicating interactions between Fe and other ions, such as Cu and Zn [13], [14], it needs to be clarified whether Fe in a specific ion module might act additively or synergistically with other ions to influence overall risk of type 2 diabetes.

Another interesting phenomenon was that both Cu and P were always ranked in the top two positions among 17 ions included in the three specific ion networks related to obesity, metabolic syndrome and type 2 diabetes, implicating potential associations of Cu and P with these metabolic disorders. Indeed, increased level of Cu was observed in diabetic patients [44]; while administration of Cu chelating reduced insulin resistance and also alleviated glucose intolerance in diabetic db/db mice [45]. In addition, high serum P level was reported to be positively associated with hypercholesterolemia and subclinical atherosclerosis [46], and might predict cardiovascular mortality in type 2 diabetes [47]. Besides its role as a single mineral, Cu also exhibits multiple mechanisms in regulating ion homeostasis under physical and pathological conditions. For examples, Cu could interact with Fe in the processes of absorption, transportation and metabolism in multiple tissues and cells, which may be modified by the amount and ratio of each ions and likewise the overall interaction might also influence whole body Cu-Fe homeostasis and related health or disease conditions [13]. Moreover, the Cu-Mo interaction was also suggested as a reaction between thiomolybdate and Cu by studies in animal model and human tissue [48], [49]. Significantly different levels of Cu-Zn-superoxide dismutase (CuZn-SOD), a form of Cu and Zn, were also reported between obese and non-obese controls [50]. In addition, the Cu/Zn ratio was reported to be associated with multiple abnormalities and diseases [51], [52], [53], [54], [55]. Regarding to P, the co-joint effect of P and Ca on increasing total mortality of middle-aged men was reported in a Sweden cohort [56], in line with adverse role of P contained ion modules in metabolic disorders.

Although current study could not provide direct evidence for the mechanistic link between plasma ions and metabolic disorders, there are plausible explanations. First, several measured ions such as Fe, Cu, Cr, Zn and Se were previously reported to be involved in the induction or defense of oxidative stress [57], [58]. Second, genetic variants of several ions were shown to be associated with diabetes. For instance, *TMPRSS6* variants were significantly associated with ferritin and risk of type 2 diabetes in Chinese [59], while ferritin itself was an independent risk factor for metabolic syndrome and type 2 diabetes [1], [2], [43]. However, we observed significantly lower intake of multiple ions in subjects with metabolic disorders (Zn, Se, Mg, Ca, P and K in overweight/obesity, Zn, Ca and P in metabolic syndrome, as well as Mg, Ca, P and K in diabetes, data not shown) in comparison with their normal counterparts, although the influence of intake levels on plasma ion concentrations remains unclear. Moreover, the strong correlations between plasma Cu levels and inflammatory markers suggest that the association between Cu levels and metabolic disorders might be partially explained by inflammatory status [60]. Therefore, the complicated network and potential mechanistic linking between multiple ions and metabolic disorders merit further investigation.

Overall, by using the novel approach of the ionomics strategy and the information theory, we observed potential links between multiple ions and metabolic abnormalities. However, since our findings were mainly driven by statistical analysis, future biological and mechanistic studies are needed to confirm the observed associations. Moreover, the cross-sectional nature of this study also prevents us from establishing causal relation. Meanwhile, the case-control design might also limit our findings to be generalized in general populations. Certainly, more prospective studies are merited in this regards. Nonetheless, our study attempts to use a different methodology to explore the associations between multiple ions and metabolic disorders in a large-scale population based study.

## Supporting Information

Table S1 **Optimum Operating Conditions for ICP-MS.**
(DOC)

Table S2 **The comparison of determined values of human serum with reference values.**
(DOC)

Table S3 **The recovery of determined elements in human plasma.**
(DOC)

Table S4 **Stability of the instrument and precision of the method.**
(DOC)

Table S5 **The exact permutation** ***P*** **values of ion combinations with overweight/obesity.**
(DOC)

Table S6 **The exact permutation** ***P*** **values of ion combinations with metabolic syndrome.**
(DOC)

Table S7 **The exact permutation** ***P*** **values of ion combinations with Type 2 diabetes.**
(DOC)

Table S8 **The overweight/obesity related ion network.**
(DOC)

Table S9 **The metabolic syndrome related ion network.**
(DOC)

Table S10 **The type 2 diabetes related ion network.**
(DOC)

Table S11 **The highly connected two ion and three ion modules.**
(DOC)
