# Supplementary material for: Associations between Ionomic Profile and Metabolic Abnormalities in Human Population
Source: PLoS One. 2012 Jun 13;7(6):e38845. doi: 10.1371/journal.pone.0038845 (PMC3374762; doi:10.1371/journal.pone.0038845)
Supplement: Table S1 — Optimum Operating Conditions for ICP-MS. (DOC) [file pone.0038845.s001.doc]

**Table S1 Optimum Operating Conditions for ICP-MS**

| Operation Parameters | |
| --- | --- |
| RF power (W) | 1550 |
| carrier gas flow (L min-1) | 0.85 |
| makeup gas flow (L min-1) | 0.43 |
| reaction gas He (mL min-1) | 4.5 |
| sample depth (mm) | 8 |
| sample uptake rate (rps) | 0.1 |
| extract 1 voltage (v) | 0 |
| extract 2 voltage (v) | -155 |
| omega bias voltage (v) | -22 |
| omega lens voltage (v) | -1 |
| octopole bias voltage (v) | -16 |
| spray chamber temperature (°C) | 2 |
| ISIS load time (s) | 15 |
| ISIS load speed (rps) | 1 |
| ISIS probe rinse time (s) | 20 |
| ISIS probe rinse speed (rps) | 0.1 |
| Data Acquisition | |
| acquisition mode | peak hopping |
| peak pattern | 3 points/peak (FullQ) |
| integration time (s) | 0.1/point |
