# Supplementary material for: Associations between Ionomic Profile and Metabolic Abnormalities in Human Population
Source: PLoS One. 2012 Jun 13;7(6):e38845. doi: 10.1371/journal.pone.0038845 (PMC3374762; doi:10.1371/journal.pone.0038845)
Supplement: Table S2 — The comparison of determined values of human serum with reference values. (DOC) [file pone.0038845.s002.doc]

**Table S2 The comparison of determined values of human serum with reference values**

| Element | Determined concentrations | Reference concentrations |
| --- | --- | --- |
| Ca (ppb) | 93.9±0.97 | 94.90 |
| Cu (ppb) | 1100.63±26.31 | 1090.00 |
| Fe (ppm) | 1.33±0.07 | 1.36 |
| K (ppb) | 166.29±7.59 | 168.30 |
| Mg (ppm) | 20.94±0.86 | 20.75 |
| Na (ppm) | 3114.65±78.75 | 3151.00 |
| Se (ppb) | 75.82±0.57 | 75.70 |
| Zn (ppb) | 1147.99±34.05 | 1132.00 |
