# Supplementary material for: Associations between Ionomic Profile and Metabolic Abnormalities in Human Population
Source: PLoS One. 2012 Jun 13;7(6):e38845. doi: 10.1371/journal.pone.0038845 (PMC3374762; doi:10.1371/journal.pone.0038845)
Supplement: Table S3 — The recovery of determined elements in human plasma. (DOC) [file pone.0038845.s003.doc]

**Table S3 The recovery of determined elements in human plasma**

| Element | Isotope monitored | Linear correlation (r) | Detection limit (ppb) | Recovery (%) |
| --- | --- | --- | --- | --- |
| Ca | 43 | 0.9999 | 1.6260 | 113.43±3.11 |
| Cr | 52 | 1.0000 | 0.0351 | 88.6±2.33 |
| Cu | 63 | 1.0000 | 0.0660 | 92.22±2.03 |
| Fe | 56 | 1.0000 | 0.1501 | 90.79±1.58 |
| K | 39 | 1.0000 | 1.1370 | 102.3±2.16 |
| Mg | 24 | 0.9999 | 0.1373 | 101.7±4.56 |
| Mn | 55 | 1.0000 | 0.0325 | 101.52±2.96 |
| Mo | 95 | 1.0000 | 0.0098 | 102.51±2.22 |
| P | 31 | 1.0000 | 2.5480 | 99.92±2.88 |
| Re | 185 | 1.0000 | 0.0000 | 82.22±2.55 |
| S | 34 | 0.9999 | 122.6000 | 112.56±5.82 |
| Sb | 121 | 1.0000 | 0.0137 | 74.87±1.63 |
| Se | 78 | 1.0000 | 0.2266 | 98.49±2.07 |
| Sn | 118 | 1.0000 | 0.0000 | 81.30±2.07 |
| Sr | 88 | 1.0000 | 0.0626 | 110.72±2.15 |
| Ti | 48 | 1.0000 | 0.0555 | 91.04±2.63 |
| Zn | 66 | 0.9999 | 0.0913 | 79.01±1.00 |
