# Supplementary material for: Associations between Ionomic Profile and Metabolic Abnormalities in Human Population
Source: PLoS One. 2012 Jun 13;7(6):e38845. doi: 10.1371/journal.pone.0038845 (PMC3374762; doi:10.1371/journal.pone.0038845)
Supplement: Table S4 — Stability of the instrument and precision of the method. (DOC) [file pone.0038845.s004.doc]

**Table S4 Stability of the instrument and precision of the method**

| Element | Stability (RSD%) of the instrument | | Precision (RSD%) of the method (n=10) |
| --- | --- | --- | --- |
|  | intraday(n=12) | interday(n=100) |  |
| Ca | 1.84 | 18.84 | 1.03 |
| Cr | 1.92 | 14.77 | 1.24 |
| Cu | 6.41 | 17.08 | 2.44 |
| Fe | 2.93 | 20.23 | 2.11 |
| K | 1.96 | 14.40 | 1.15 |
| Mg | 2.70 | 15.13 | 1.06 |
| Mn | 1.85 | 14.60 | 1.19 |
| Mo | 2.03 | 14.05 | 2.26 |
| P | 2.69 | 14.98 | 2.55 |
| Re | 1.19 | 14.53 | 0.73 |
| S | 1.99 | 14.42 | 1.36 |
| Sb | 1.71 | 13.61 | 0.99 |
| Se | 3.20 | 13.60 | 1.68 |
| Sn | 4.77 | 13.76 | 1.65 |
| Sr | 1.94 | 13.81 | 0.28 |
| Ti | 1.91 | 14.12 | 0.88 |
| Zn | 1.50 | 16.52 | 0.90 |
