# Supplementary material for: Associations between Ionomic Profile and Metabolic Abnormalities in Human Population
Source: PLoS One. 2012 Jun 13;7(6):e38845. doi: 10.1371/journal.pone.0038845 (PMC3374762; doi:10.1371/journal.pone.0038845)
Supplement: Table S7 — The exact permutation P values of ion combinations with Type 2 diabetes. (DOC) [file pone.0038845.s007.doc]

**Table S7 The exact permutation *P* values of ion combinations with Type 2 diabetes**

|  | Number of ions in the combination | Exact permutation *P* value |
| --- | --- | --- |
| Cr | 1 | 0.001998002 |
| K | 1 | 0.00999001 |
| Fe_Sr | 2 | 0.000999001 |
| Sr_Re | 2 | 0.001998002 |
| Zn_Re | 2 | 0.006993007 |
| Cu_Mo | 2 | 0.006993007 |
| Cu_P | 2 | 0.007992008 |
| Cu_Mo_P | 3 | 0.000999001 |
| Cr_Mn_Cu | 3 | 0.001998002 |
| Cr_Cu_P | 3 | 0.001998002 |
| Zn_Ca_Re | 3 | 0.001998002 |
| Fe_Zn_S | 3 | 0.002997003 |
| Fe_Mo_Sr | 3 | 0.002997003 |
| Fe_Sr_P | 3 | 0.003996004 |
| Sn_Ti_P | 3 | 0.003996004 |
| Cr_Ca_P | 3 | 0.004995005 |
| Mn_Fe_Sr | 3 | 0.005994006 |
| Fe_Ca_P | 3 | 0.005994006 |
| Cu_Sn_Re | 3 | 0.005994006 |
| Sn_Sb_P | 3 | 0.005994006 |
| Fe_Zn_Mg | 3 | 0.006993007 |
| Cu_Sr_Sb | 3 | 0.006993007 |
| Fe_Zn_Sb | 3 | 0.007992008 |
| Cu_Sn_P | 3 | 0.007992008 |
| Mn_Fe_S | 3 | 0.008991009 |
| Cr_Fe_Zn_S | 4 | 0.000999001 |
| Cr_Sn_Sb_S | 4 | 0.000999001 |
| Mn_Fe_P_Re | 4 | 0.000999001 |
| Mn_Se_Sn_P | 4 | 0.000999001 |
| Mn_Sr_Sb_Ca | 4 | 0.000999001 |
| Fe_Zn_Mo_S | 4 | 0.000999001 |
| Fe_Mo_Sr_Re | 4 | 0.000999001 |
| Cu_Mo_Sr_Sn | 4 | 0.000999001 |
| Cu_Sn_P_Re | 4 | 0.000999001 |
| Cr_Se_Ti_P | 4 | 0.001998002 |
| Cr_Mo_Sb_P | 4 | 0.001998002 |
| Mn_Fe_Zn_Sn | 4 | 0.001998002 |
| Mn_Fe_Sn_P | 4 | 0.001998002 |
| Mn_Cu_Sr_P | 4 | 0.001998002 |
| Cr_Fe_Zn_Re | 4 | 0.002997003 |
| Mn_Cu_Mo_P | 4 | 0.002997003 |
| Mn_Cu_P_K | 4 | 0.002997003 |
| Cr_Cu_Ca_K | 4 | 0.003996004 |
| Mn_Fe_Mo_Sr | 4 | 0.003996004 |
| Mn_Fe_Sb_P | 4 | 0.003996004 |
| Mn_Fe_Mg_P | 4 | 0.003996004 |
| Mn_Zn_Sn_Re | 4 | 0.003996004 |
| Mn_Cu_Sn_P | 4 | 0.003996004 |
| Mn_Mo_P_S | 4 | 0.003996004 |
| Fe_Zn_Sr_Ca | 4 | 0.003996004 |
| Cu_Mo_Mg_P | 4 | 0.003996004 |
| Cu_Sn_Sb_P | 4 | 0.003996004 |
| Fe_Cu_Sr_P | 4 | 0.004995005 |
| Cu_Sn_Sb_Mg | 4 | 0.004995005 |
| Mo_Mg_P_S | 4 | 0.004995005 |
| Cr_Cu_Sn_P | 4 | 0.005994006 |
| Mn_Cu_Sb_P | 4 | 0.005994006 |
| Mo_Mg_Ca_K | 4 | 0.005994006 |
| Cr_Cu_Mo_P | 4 | 0.006993007 |
| Zn_Cu_Mo_P | 4 | 0.006993007 |
| Cr_Fe_Cu_Se | 4 | 0.007992008 |
| Mn_Se_Ti_P | 4 | 0.007992008 |
| Fe_Se_P_Re | 4 | 0.007992008 |
| Cr_Sr_Ca_P | 4 | 0.008991009 |
| Fe_Cu_Mo_Sn | 4 | 0.008991009 |
| Mn_Fe_Sr_K | 4 | 0.00999001 |
| Mn_Sb_Ti_P | 4 | 0.00999001 |
| Fe_Mo_Sr_Sn | 4 | 0.00999001 |
| Cu_Sb_Mg_P | 4 | 0.00999001 |
| Cr_Mn_Fe_Cu_Re | 5 | 0.000999001 |
| Cr_Mn_Cu_Mo_P | 5 | 0.000999001 |
| Cr_Mn_Se_Ti_P | 5 | 0.000999001 |
| Cr_Cu_Sb_Mg_P | 5 | 0.000999001 |
| Mn_Fe_Zn_Sr_Sn | 5 | 0.000999001 |
| Mn_Fe_Zn_Sr_Re | 5 | 0.000999001 |
| Mn_Cu_Sn_P_Re | 5 | 0.000999001 |
| Mn_Sn_Mg_P_S | 5 | 0.000999001 |
| Fe_Zn_Se_Ti_P | 5 | 0.000999001 |
| Fe_Mo_Sr_Sb_Ti | 5 | 0.000999001 |
| Cr_Mn_Fe_Cu_P | 5 | 0.001998002 |
| Cr_Mn_Cu_Sr_P | 5 | 0.001998002 |
| Cr_Mn_Cu_Sr_K | 5 | 0.001998002 |
| Cr_Cu_Sn_P_Re | 5 | 0.001998002 |
| Mn_Fe_Mo_Sn_P | 5 | 0.001998002 |
| Mn_Cu_Mo_Sr_Sn | 5 | 0.001998002 |
| Mn_Cu_Sb_Mg_P | 5 | 0.001998002 |
| Cr_Sn_Mg_P_S | 5 | 0.002997003 |
| Mn_Fe_Mg_P_S | 5 | 0.002997003 |
| Mn_Cu_Sr_Sn_P | 5 | 0.002997003 |
| Fe_Zn_Cu_Ti_P | 5 | 0.002997003 |
| Cr_Fe_Sb_P_S | 5 | 0.003996004 |
| Cr_Cu_Sr_Sn_P | 5 | 0.003996004 |
| Cr_Cu_Sn_Sb_P | 5 | 0.003996004 |
| Mn_Zn_Ti_Ca_Re | 5 | 0.003996004 |
| Mn_Cu_Mo_Sb_Ti | 5 | 0.003996004 |
| Mn_Se_Sn_Sb_Mg | 5 | 0.003996004 |
| Fe_Zn_Mo_S_Re | 5 | 0.003996004 |
| Mo_Sn_Ti_Mg_P | 5 | 0.003996004 |
| Cr_Mn_Cu_Sb_Ti | 5 | 0.004995005 |
| Cr_Cu_Mo_Sr_Sn | 5 | 0.004995005 |
| Mn_Cu_Mo_Sn_P | 5 | 0.004995005 |
| Mn_Cu_Sn_Sb_P | 5 | 0.004995005 |
| Fe_Cu_Sr_Ca_Re | 5 | 0.004995005 |
| Mn_Fe_Zn_Sb_Re | 5 | 0.005994006 |
| Fe_Zn_Mg_P_Re | 5 | 0.005994006 |
| Fe_Mo_Ti_Mg_P | 5 | 0.005994006 |
| Cr_Mn_Fe_Zn_Sr | 5 | 0.006993007 |
| Mn_Fe_Sb_P_S | 5 | 0.006993007 |
| Fe_Cu_Mo_Sr_Sn | 5 | 0.006993007 |
| Mn_Fe_Zn_Sr_Ca | 5 | 0.007992008 |
| Mn_Cu_Se_Mo_Sb | 5 | 0.007992008 |
| Fe_Cu_Se_Sr_P | 5 | 0.007992008 |
| Fe_Se_Sb_P_Re | 5 | 0.007992008 |
| Zn_Se_Sb_Mg_P | 5 | 0.007992008 |
| Cr_Fe_Mo_Ca_P | 5 | 0.008991009 |
| Cr_Fe_Sn_Ca_K | 5 | 0.008991009 |
| Cr_Mo_Sb_P_S | 5 | 0.008991009 |
| Fe_Cu_Sn_Ca_P | 5 | 0.008991009 |
| Fe_Se_Sr_K_Re | 5 | 0.008991009 |
| Fe_Mo_Sn_Sb_P | 5 | 0.008991009 |
| Zn_Cu_Mo_Sr_P | 5 | 0.008991009 |
| Zn_Mo_Sr_Sn_P | 5 | 0.008991009 |
| Cr_Zn_Sn_Sb_K | 5 | 0.00999001 |
| Cr_Cu_Se_Mo_Re | 5 | 0.00999001 |
| Mn_Cu_Sr_P_K | 5 | 0.00999001 |
| Mn_Mo_Sb_P_S | 5 | 0.00999001 |
| Zn_Mo_Sr_Ti_Ca | 5 | 0.00999001 |
| Cr_Mn_Cu_Sr_P_K | 6 | 0.000999001 |
| Cr_Mn_Cu_Sn_P_Re | 6 | 0.000999001 |
| Cr_Cu_Mo_Sr_P_K | 6 | 0.000999001 |
| Zn_Cu_Sn_Mg_Ca_S | 6 | 0.000999001 |
| Cr_Cu_Mo_Sn_Sb_Mg | 6 | 0.001998002 |
| Mn_Zn_Se_Sn_Sb_P | 6 | 0.001998002 |
| Zn_Cu_Mo_Mg_P_S | 6 | 0.001998002 |
| Zn_Cu_Sr_Sb_Ca_P | 6 | 0.001998002 |
| Cr_Mn_Sn_Mg_P_S | 6 | 0.002997003 |
| Cr_Fe_Zn_Mo_S_Re | 6 | 0.002997003 |
| Cr_Fe_Mo_Sn_Sb_P | 6 | 0.002997003 |
| Cr_Fe_Mo_Sb_Ti_P | 6 | 0.002997003 |
| Mn_Mo_Sn_Mg_P_S | 6 | 0.002997003 |
| Fe_Zn_Sr_Sn_Ca_Re | 6 | 0.002997003 |
| Fe_Se_Sn_Sb_Ca_K | 6 | 0.002997003 |
| Cr_Mn_Cu_Sn_Sb_P | 6 | 0.003996004 |
| Cr_Zn_Se_Sn_Mg_K | 6 | 0.003996004 |
| Cr_Mo_Sb_Mg_Ca_P | 6 | 0.003996004 |
| Mn_Cu_Sr_Sn_P_Re | 6 | 0.003996004 |
| Mn_Cu_Sn_Sb_P_S | 6 | 0.003996004 |
| Fe_Cu_Mo_Sr_Ti_Re | 6 | 0.003996004 |
| Zn_Sr_Sn_Sb_Ca_P | 6 | 0.003996004 |
| Cr_Fe_Se_Mo_Mg_P | 6 | 0.004995005 |
| Cr_Fe_Se_Sr_K_Re | 6 | 0.004995005 |
| Cr_Cu_Se_Sn_Mg_P | 6 | 0.004995005 |
| Cr_Cu_Mo_Sr_Sn_Re | 6 | 0.004995005 |
| Cr_Cu_Mo_Sn_Sb_Ti | 6 | 0.004995005 |
| Cr_Cu_Mo_Sn_Mg_P | 6 | 0.004995005 |
| Cr_Cu_Sn_Sb_P_Re | 6 | 0.004995005 |
| Cr_Cu_Sb_P_K_Re | 6 | 0.004995005 |
| Cr_Mo_Sn_Sb_Ti_P | 6 | 0.004995005 |
| Fe_Cu_Se_Sn_Mg_P | 6 | 0.004995005 |
| Zn_Cu_Se_Mo_P_S | 6 | 0.004995005 |
| Cu_Mo_Sn_Mg_P_S | 6 | 0.004995005 |
| Cu_Sr_Sb_Ca_P_Re | 6 | 0.004995005 |
| Cu_Sr_Sb_P_K_Re | 6 | 0.004995005 |
| Cr_Mn_Fe_Cu_P_Re | 6 | 0.005994006 |
| Cr_Mn_Cu_Mo_Sr_Re | 6 | 0.005994006 |
| Cr_Fe_Zn_Se_Ti_P | 6 | 0.005994006 |
| Cr_Cu_Sb_Ca_K_Re | 6 | 0.005994006 |
| Mn_Fe_Zn_Cu_Mg_Ca | 6 | 0.005994006 |
| Cr_Mn_Cu_Mo_Sr_Sn | 6 | 0.006993007 |
| Cr_Fe_Cu_Sb_Ca_P | 6 | 0.006993007 |
| Cr_Fe_Mo_Ti_Ca_P | 6 | 0.006993007 |
| Cr_Zn_Mo_Mg_P_S | 6 | 0.006993007 |
| Cr_Cu_Mo_Sr_Ti_Mg | 6 | 0.006993007 |
| Cr_Cu_Mo_Sr_Mg_P | 6 | 0.006993007 |
| Cr_Cu_Sr_Sb_Mg_K | 6 | 0.006993007 |
| Mn_Fe_Sn_Sb_P_Re | 6 | 0.006993007 |
| Mn_Zn_Se_Mo_Mg_P | 6 | 0.006993007 |
| Mn_Cu_Se_Sr_Sn_Ti | 6 | 0.006993007 |
| Fe_Zn_Cu_P_S_Re | 6 | 0.006993007 |
| Fe_Cu_Sr_Sn_Sb_Re | 6 | 0.006993007 |
| Fe_Cu_Mo_Sn_Ca_P | 6 | 0.007992008 |
| Mn_Fe_Se_Ti_P_K | 6 | 0.008991009 |
| Mn_Cu_Se_Sr_Sb_S | 6 | 0.008991009 |
| Mn_Cu_Mo_Sb_P_S | 6 | 0.008991009 |
| Fe_Zn_Cu_Ti_P_Re | 6 | 0.008991009 |
| Fe_Cu_Sr_Sb_P_S | 6 | 0.008991009 |
| Fe_Cu_Sn_Sb_Ca_S | 6 | 0.008991009 |
| Fe_Se_Sn_Ti_S_K | 6 | 0.008991009 |
| Cr_Mn_Zn_Mo_Mg_S | 6 | 0.00999001 |
| Cr_Mn_Cu_Mo_Sb_Ti | 6 | 0.00999001 |
| Cr_Cu_Se_Mo_Mg_S | 6 | 0.00999001 |
| Cr_Cu_Mo_Sb_P_S | 6 | 0.00999001 |
| Cr_Sr_Sb_Ti_Ca_P | 6 | 0.00999001 |
| Mn_Fe_Zn_Mo_Sn_Re | 6 | 0.00999001 |
| Mn_Cu_Sr_P_K_Re | 6 | 0.00999001 |
| Fe_Cu_Mo_Sr_Mg_P | 6 | 0.00999001 |
| Zn_Sr_Sn_Sb_Ca_K | 6 | 0.00999001 |
| Cr_Fe_Cu_Sr_Sb_P_S | 7 | 0.000999001 |
| Cr_Zn_Cu_Mo_Mg_P_S | 7 | 0.000999001 |
| Cr_Zn_Cu_Sr_Sb_Ca_P | 7 | 0.000999001 |
| Cr_Zn_Mo_Mg_P_S_Re | 7 | 0.000999001 |
| Cr_Cu_Mo_Sn_Mg_P_S | 7 | 0.000999001 |
| Mn_Zn_Cu_Sn_Mg_Ca_S | 7 | 0.000999001 |
| Mn_Mo_Sr_Sb_Mg_P_Re | 7 | 0.000999001 |
| Fe_Zn_Cu_Mo_Sb_Mg_P | 7 | 0.000999001 |
| Fe_Cu_Se_Sr_Sn_Ti_P | 7 | 0.000999001 |
| Fe_Se_Mo_Sn_Sb_Ca_K | 7 | 0.000999001 |
| Cr_Fe_Cu_Mo_Sr_Sn_Re | 7 | 0.001998002 |
| Mn_Fe_Zn_Cu_Mo_P_Re | 7 | 0.001998002 |
| Fe_Zn_Sn_Mg_P_S_Re | 7 | 0.001998002 |
| Cr_Mn_Cu_Mo_Sn_Sb_Ti | 7 | 0.002997003 |
| Cr_Fe_Cu_Mo_Mg_P_Re | 7 | 0.002997003 |
| Cr_Cu_Mo_Sr_Sb_Mg_P | 7 | 0.002997003 |
| Mn_Fe_Zn_Sn_Sb_Ca_P | 7 | 0.002997003 |
| Mn_Cu_Sr_Sn_Mg_P_Re | 7 | 0.002997003 |
| Fe_Zn_Cu_Mo_Sn_Sb_Re | 7 | 0.002997003 |
| Fe_Cu_Sn_Mg_Ca_P_S | 7 | 0.002997003 |
| Cr_Mn_Fe_Zn_Sb_Ca_Re | 7 | 0.003996004 |
| Cr_Mn_Cu_Sb_Mg_P_Re | 7 | 0.003996004 |
| Cr_Fe_Cu_Se_Mo_Sb_P | 7 | 0.003996004 |
| Cr_Zn_Cu_Se_Sn_Sb_P | 7 | 0.003996004 |
| Cr_Zn_Cu_Sn_Mg_Ca_S | 7 | 0.003996004 |
| Mn_Fe_Zn_Se_Ti_P_Re | 7 | 0.003996004 |
| Mn_Fe_Se_Sn_Sb_P_Re | 7 | 0.003996004 |
| Mn_Cu_Mo_Sr_Mg_P_K | 7 | 0.003996004 |
| Mn_Cu_Mo_Sb_P_S_Re | 7 | 0.003996004 |
| Cr_Mn_Fe_Se_Sn_P_Re | 7 | 0.004995005 |
| Cr_Mn_Cu_Mo_Sr_Sb_S | 7 | 0.004995005 |
| Cr_Mn_Sr_Sb_Ti_Ca_P | 7 | 0.004995005 |
| Mn_Fe_Zn_Se_Sn_Sb_P | 7 | 0.004995005 |
| Mn_Zn_Cu_Se_Sn_Ca_S | 7 | 0.004995005 |
| Fe_Zn_Mo_Sn_Ca_P_Re | 7 | 0.004995005 |
| Cr_Mn_Fe_Se_Mo_Mg_P | 7 | 0.005994006 |
| Cr_Fe_Cu_Se_Sn_Sb_P | 7 | 0.005994006 |
| Cr_Fe_Se_Mo_Mg_P_Re | 7 | 0.005994006 |
| Cr_Fe_Se_Sn_Sb_P_Re | 7 | 0.005994006 |
| Cr_Zn_Mo_Mg_S_K_Re | 7 | 0.005994006 |
| Cr_Cu_Sb_Mg_Ca_P_Re | 7 | 0.005994006 |
| Fe_Zn_Cu_Sn_Ti_Ca_Re | 7 | 0.005994006 |
| Fe_Cu_Mo_Sb_Mg_Ca_P | 7 | 0.005994006 |
| Cr_Fe_Zn_Cu_Se_Sr_P | 7 | 0.006993007 |
| Cr_Fe_Zn_Cu_Mg_P_Re | 7 | 0.006993007 |
| Cr_Fe_Cu_Mo_Mg_P_S | 7 | 0.006993007 |
| Cr_Zn_Cu_Se_Sr_Sn_P | 7 | 0.006993007 |
| Cr_Cu_Se_Sr_Sn_Sb_Ti | 7 | 0.006993007 |
| Zn_Cu_Se_Mo_Sn_P_S | 7 | 0.006993007 |
| Cr_Fe_Cu_Mo_Sr_Sb_Re | 7 | 0.007992008 |
| Cr_Zn_Cu_Mo_Sr_Sb_P | 7 | 0.007992008 |
| Mn_Se_Mo_Sb_P_K_Re | 7 | 0.007992008 |
| Fe_Cu_Se_Sn_Sb_Ca_P | 7 | 0.007992008 |
| Fe_Se_Sn_Sb_Ca_K_Re | 7 | 0.007992008 |
| Zn_Mo_Ti_Mg_P_S_Re | 7 | 0.007992008 |
| Cr_Mn_Sn_Sb_Mg_P_S | 7 | 0.008991009 |
| Cr_Fe_Zn_Cu_Sr_Sn_Re | 7 | 0.008991009 |
| Mn_Fe_Zn_Se_Sb_Ti_P | 7 | 0.008991009 |
| Mn_Fe_Mo_Sn_Sb_Mg_P | 7 | 0.008991009 |
| Mn_Cu_Se_Sn_Ca_K_Re | 7 | 0.008991009 |
| Mn_Cu_Sn_Sb_P_S_Re | 7 | 0.008991009 |
| Mn_Mo_Sn_Ti_Mg_P_S | 7 | 0.008991009 |
| Fe_Cu_Mo_Sr_Sb_P_S | 7 | 0.008991009 |
| Fe_Se_Sn_Ti_Mg_K_Re | 7 | 0.008991009 |
| Zn_Cu_Sn_Mg_Ca_P_S | 7 | 0.008991009 |
| Cr_Fe_Zn_Se_Sn_Sb_P | 7 | 0.00999001 |
| Cr_Fe_Zn_Se_Sb_Ti_P | 7 | 0.00999001 |
| Mn_Fe_Zn_Cu_Sn_Ca_P | 7 | 0.00999001 |
| Mn_Zn_Cu_Se_Sn_Ti_P | 7 | 0.00999001 |
| Mn_Zn_Mo_Sr_Mg_S_K | 7 | 0.00999001 |
| Mn_Zn_Mo_Mg_S_K_Re | 7 | 0.00999001 |
| Mn_Cu_Se_Sr_Sb_S_Re | 7 | 0.00999001 |
| Fe_Cu_Mo_Sr_Mg_P_Re | 7 | 0.00999001 |
| Fe_Cu_Sr_Sb_P_S_Re | 7 | 0.00999001 |
| Cr_Zn_Cu_Se_Sn_Sb_P_Re | 8 | 0.000999001 |
| Fe_Zn_Mo_Sn_Mg_P_S_Re | 8 | 0.000999001 |
| Fe_Cu_Se_Sr_Sn_Sb_P_S | 8 | 0.000999001 |
| Cr_Mn_Fe_Mo_Sb_Ti_P_S | 8 | 0.001998002 |
| Cr_Fe_Cu_Mo_Sr_Sb_P_S | 8 | 0.001998002 |
| Cr_Fe_Se_Mo_Sn_Sb_Mg_P | 8 | 0.001998002 |
| Mn_Fe_Cu_Sn_Sb_P_S_Re | 8 | 0.001998002 |
| Cr_Mn_Fe_Cu_Se_Mo_Sb_P | 8 | 0.002997003 |
| Cr_Fe_Zn_Cu_Mo_Sr_Sb_Ti | 8 | 0.002997003 |
| Cr_Zn_Mo_Ti_Mg_P_S_Re | 8 | 0.002997003 |
| Cr_Cu_Se_Mo_Sr_Mg_P_S | 8 | 0.002997003 |
| Mn_Fe_Cu_Mo_Sr_Sb_P_S | 8 | 0.002997003 |
| Cr_Mn_Cu_Sr_Sb_P_K_Re | 8 | 0.003996004 |
| Cr_Fe_Se_Mo_Sr_Ca_P_K | 8 | 0.003996004 |
| Cr_Zn_Cu_Sn_Mg_Ca_S_Re | 8 | 0.003996004 |
| Mn_Fe_Cu_Se_Mo_Sb_P_S | 8 | 0.003996004 |
| Mn_Zn_Cu_Mo_Mg_P_S_Re | 8 | 0.003996004 |
| Fe_Se_Mo_Sn_Ca_P_S_K | 8 | 0.003996004 |
| Zn_Cu_Mo_Sn_Mg_Ca_S_Re | 8 | 0.003996004 |
| Zn_Cu_Sr_Sn_Ti_Mg_P_S | 8 | 0.003996004 |
| Cu_Sr_Sn_Sb_Mg_P_S_K | 8 | 0.003996004 |
| Cr_Mn_Fe_Se_Sr_Sn_S_K | 8 | 0.004995005 |
| Cr_Mn_Fe_Se_Sn_Sb_Ca_K | 8 | 0.004995005 |
| Cr_Mn_Cu_Mo_Sr_Sb_Ti_Re | 8 | 0.004995005 |
| Cr_Mn_Sr_Sb_Mg_P_S_Re | 8 | 0.004995005 |
| Cr_Zn_Cu_Se_Mo_Sn_P_S | 8 | 0.004995005 |
| Mn_Fe_Cu_Sn_Sb_P_K_Re | 8 | 0.004995005 |
| Mn_Cu_Mo_Sr_Sb_P_K_Re | 8 | 0.004995005 |
| Cu_Sr_Sn_Sb_Mg_P_S_Re | 8 | 0.004995005 |
| Cr_Mn_Fe_Zn_Cu_Se_S_Re | 8 | 0.005994006 |
| Cr_Mn_Fe_Cu_Se_Mo_Mg_P | 8 | 0.005994006 |
| Cr_Fe_Cu_Se_Mo_Sb_Mg_S | 8 | 0.005994006 |
| Cr_Zn_Cu_Mo_Sb_Mg_Ca_P | 8 | 0.005994006 |
| Cr_Zn_Cu_Mo_Mg_Ca_P_Re | 8 | 0.005994006 |
| Cr_Cu_Se_Mo_Sr_Sb_Ti_Mg | 8 | 0.005994006 |
| Fe_Zn_Cu_Mo_Sb_Mg_P_Re | 8 | 0.005994006 |
| Cr_Mn_Fe_Cu_Se_Sn_Sb_P | 8 | 0.006993007 |
| Cr_Mn_Fe_Cu_Mo_Mg_P_S | 8 | 0.006993007 |
| Cr_Mn_Fe_Cu_Sb_P_S_Re | 8 | 0.006993007 |
| Cr_Cu_Mo_Sn_Mg_Ca_P_S | 8 | 0.006993007 |
| Mn_Fe_Cu_Mo_Sr_Sb_Mg_P | 8 | 0.006993007 |
| Mn_Fe_Cu_Mo_Sb_Mg_P_S | 8 | 0.006993007 |
| Mn_Mo_Sn_Ti_Mg_P_S_Re | 8 | 0.006993007 |
| Cr_Mn_Zn_Mo_Mg_S_K_Re | 8 | 0.007992008 |
| Cr_Fe_Zn_Cu_Se_Ca_P_Re | 8 | 0.007992008 |
| Cr_Fe_Cu_Mo_Sb_Mg_P_S | 8 | 0.007992008 |
| Cr_Fe_Mo_Sb_Mg_P_K_Re | 8 | 0.007992008 |
| Cr_Cu_Mo_Sb_Ti_Mg_P_S | 8 | 0.007992008 |
| Mn_Zn_Cu_Se_Sn_P_S_Re | 8 | 0.007992008 |
| Mn_Cu_Se_Sr_Mg_Ca_P_K | 8 | 0.007992008 |
| Fe_Zn_Cu_Mo_Mg_Ca_P_K | 8 | 0.007992008 |
| Fe_Cu_Se_Sn_Ti_Ca_P_Re | 8 | 0.007992008 |
| Fe_Cu_Mo_Sr_Sn_Sb_P_S | 8 | 0.007992008 |
| Zn_Sr_Sn_Sb_Mg_P_S_K | 8 | 0.007992008 |
| Cr_Mn_Fe_Zn_Cu_Sn_Ti_Ca | 8 | 0.008991009 |
| Cr_Mn_Fe_Zn_Sn_Ca_P_Re | 8 | 0.008991009 |
| Cr_Mn_Fe_Cu_Se_Sb_P_S | 8 | 0.008991009 |
| Cr_Cu_Mo_Sn_Mg_P_S_Re | 8 | 0.008991009 |
| Mn_Zn_Cu_Se_Sn_Sb_P_Re | 8 | 0.008991009 |
| Zn_Sr_Sb_Ti_Mg_P_K_Re | 8 | 0.008991009 |
| Cr_Mn_Fe_Se_Sn_Sb_P_Re | 8 | 0.00999001 |
| Cr_Mn_Zn_Mo_Ti_P_S_Re | 8 | 0.00999001 |
| Cr_Fe_Cu_Se_Mo_Sn_Mg_P | 8 | 0.00999001 |
| Cr_Zn_Cu_Ti_Mg_P_S_Re | 8 | 0.00999001 |
| Cr_Cu_Sr_Sb_Mg_Ca_P_Re | 8 | 0.00999001 |
| Mn_Fe_Zn_Sr_Sn_Ti_Ca_K | 8 | 0.00999001 |
| Mn_Fe_Se_Mo_Sn_Sb_Ca_K | 8 | 0.00999001 |
| Mn_Zn_Mo_Sr_Sn_Ti_Mg_Re | 8 | 0.00999001 |
| Mn_Cu_Se_Mo_Sn_Sb_P_S | 8 | 0.00999001 |
| Mn_Cu_Sr_Sn_Sb_Mg_P_Re | 8 | 0.00999001 |
| Fe_Se_Mo_Ca_P_S_K_Re | 8 | 0.00999001 |
| Cu_Mo_Sr_Sb_Mg_P_S_K | 8 | 0.00999001 |
| Cu_Sr_Sb_Mg_Ca_P_K_Re | 8 | 0.00999001 |

* Exact permutation *P*<0.01 were showed only.
