# Supplementary material for: Associations between Ionomic Profile and Metabolic Abnormalities in Human Population
Source: PLoS One. 2012 Jun 13;7(6):e38845. doi: 10.1371/journal.pone.0038845 (PMC3374762; doi:10.1371/journal.pone.0038845)
Supplement: Table S8 — The overweight/obesity related ion network. (DOC) [file pone.0038845.s008.doc]

**Table S8 The overweight/obesity related ion network**

| **Ion 1** | **Ion 2** | **Fisher score of edge** |
| --- | --- | --- |
| Cu | P | 1063.948236 |
| Cu | Mo | 939.5906499 |
| P | Mo | 898.1381212 |
| Cr | Cu | 884.3206116 |
| Cu | Sb | 884.3206116 |
| Cr | P | 842.868083 |
| P | Sb | 842.868083 |
| Cu | Sn | 815.2330638 |
| P | Sn | 773.7805352 |
| Cu | Mg | 773.7805352 |
| Cu | Re | 746.1455161 |
| P | Mg | 732.3280065 |
| Cu | Fe | 732.3280065 |
| Cu | Zn | 732.3280065 |
| Cu | S | 732.3280065 |
| Cr | Mo | 718.5104969 |
| Mo | Sb | 718.5104969 |
| Cu | Mn | 718.5104969 |
| P | Re | 704.6929874 |
| P | Fe | 690.8754778 |
| P | Zn | 690.8754778 |
| P | S | 690.8754778 |
| Mn | P | 677.0579683 |
| Cr | Sb | 663.2404587 |
| Cu | Sr | 663.2404587 |
| Mo | Sn | 649.4229492 |
| P | Sr | 621.78793 |
| Cu | Se | 621.78793 |
| Mo | Mg | 607.9704205 |
| Cu | Ti | 607.9704205 |
| Cu | Ca | 607.9704205 |
| Cr | Sn | 594.1529109 |
| Sn | Sb | 594.1529109 |
| P | Se | 580.3354014 |
| Re | Mo | 580.3354014 |
| P | Ti | 566.5178918 |
| P | Ca | 566.5178918 |
| Cu | K | 566.5178918 |
| Fe | Mo | 566.5178918 |
| Zn | Mo | 566.5178918 |
| S | Mo | 566.5178918 |
| Cr | Mg | 552.7003823 |
| Mn | Mo | 552.7003823 |
| Mg | Sb | 552.7003823 |
| P | K | 525.0653632 |
| Cr | Re | 525.0653632 |
| Re | Sb | 525.0653632 |
| Cr | Fe | 511.2478536 |
| Cr | Zn | 511.2478536 |
| Cr | S | 511.2478536 |
| Fe | Sb | 511.2478536 |
| Zn | Sb | 511.2478536 |
| S | Sb | 511.2478536 |
| Mo | Sr | 497.430344 |
| Cr | Mn | 497.430344 |
| Mn | Sb | 497.430344 |
| Sn | Mg | 483.6128345 |
| Mo | Se | 455.9778154 |
| Re | Sn | 455.9778154 |
| Mo | Ti | 442.1603058 |
| Mo | Ca | 442.1603058 |
| Cr | Sr | 442.1603058 |
| Sr | Sb | 442.1603058 |
| Fe | Sn | 442.1603058 |
| Zn | Sn | 442.1603058 |
| S | Sn | 442.1603058 |
| Mn | Sn | 428.3427963 |
| Re | Mg | 414.5252867 |
