# Supplementary material for: Associations between Ionomic Profile and Metabolic Abnormalities in Human Population
Source: PLoS One. 2012 Jun 13;7(6):e38845. doi: 10.1371/journal.pone.0038845 (PMC3374762; doi:10.1371/journal.pone.0038845)
Supplement: Table S9 — The metabolic syndrome related ion network. (DOC) [file pone.0038845.s009.doc]

**Table S9 The metabolic syndrome related ion network**

| **Ion 1** | **Ion 2** | **Fisher score of edge** |
| --- | --- | --- |
| P | Cu | 1022.495707 |
| P | Mo | 994.8606881 |
| P | Zn | 994.8606881 |
| P | Mn | 994.8606881 |
| Cr | P | 953.4081594 |
| P | Sn | 925.7731403 |
| P | Mg | 925.7731403 |
| P | Fe | 898.1381212 |
| P | S | 884.3206116 |
| P | Sb | 884.3206116 |
| P | Re | 842.868083 |
| P | Sr | 815.2330638 |
| P | Se | 801.4155543 |
| P | Ti | 773.7805352 |
| P | K | 759.9630256 |
| Cu | Mo | 746.1455161 |
| Cu | Zn | 746.1455161 |
| Cu | Mn | 746.1455161 |
| P | Ca | 732.3280065 |
| Mo | Zn | 718.5104969 |
| Mo | Mn | 718.5104969 |
| Zn | Mn | 718.5104969 |
| Cr | Cu | 704.6929874 |
| Cr | Mo | 677.0579683 |
| Cr | Zn | 677.0579683 |
| Cr | Mn | 677.0579683 |
| Cu | Sn | 677.0579683 |
| Cu | Mg | 677.0579683 |
| Cu | Fe | 649.4229492 |
| Mo | Sn | 649.4229492 |
| Mo | Mg | 649.4229492 |
| Zn | Sn | 649.4229492 |
| Zn | Mg | 649.4229492 |
| Sn | Mn | 649.4229492 |
| Mn | Mg | 649.4229492 |
| Cu | S | 635.6054396 |
| Cu | Sb | 635.6054396 |
| Mo | Fe | 621.78793 |
| Fe | Zn | 621.78793 |
| Fe | Mn | 621.78793 |
| Cr | Sn | 607.9704205 |
| Cr | Mg | 607.9704205 |
| Mo | S | 607.9704205 |
| Mo | Sb | 607.9704205 |
| Zn | S | 607.9704205 |
| Zn | Sb | 607.9704205 |
| S | Mn | 607.9704205 |
| Mn | Sb | 607.9704205 |
| Cu | Re | 594.1529109 |
| Cr | Fe | 580.3354014 |
| Sn | Mg | 580.3354014 |
| Cr | S | 566.5178918 |
| Cr | Sb | 566.5178918 |
| Cu | Sr | 566.5178918 |
| Mo | Re | 566.5178918 |
| Zn | Re | 566.5178918 |
| Mn | Re | 566.5178918 |
| Cu | Se | 552.7003823 |
| Fe | Sn | 552.7003823 |
| Fe | Mg | 552.7003823 |
| Mo | Sr | 538.8828727 |
| Zn | Sr | 538.8828727 |
| S | Sn | 538.8828727 |
| S | Mg | 538.8828727 |
| Sr | Mn | 538.8828727 |
| Sn | Sb | 538.8828727 |
| Mg | Sb | 538.8828727 |
| Cr | Re | 525.0653632 |
