# Supplementary material for: Associations between Ionomic Profile and Metabolic Abnormalities in Human Population
Source: PLoS One. 2012 Jun 13;7(6):e38845. doi: 10.1371/journal.pone.0038845 (PMC3374762; doi:10.1371/journal.pone.0038845)
Supplement: Table S10 — The type 2 diabetes related ion network. (DOC) [file pone.0038845.s010.doc]

**Table S10 The type 2 diabetes related ion network**

| **Ion 1** | **Ion 2** | **Fisher score of edge** |
| --- | --- | --- |
| Cu | P | 607.9704205 |
| Fe | P | 552.7003823 |
| P | Sn | 552.7003823 |
| P | Cr | 538.8828727 |
| Sr | P | 525.0653632 |
| Mo | P | 525.0653632 |
| P | Mn | 525.0653632 |
| P | Zn | 511.2478536 |
| Fe | Cu | 497.430344 |
| Cu | Sn | 497.430344 |
| P | S | 497.430344 |
| Cu | Cr | 483.6128345 |
| P | Sb | 483.6128345 |
| P | Re | 483.6128345 |
| Sr | Cu | 469.7953249 |
| Cu | Mo | 469.7953249 |
| Cu | Mn | 469.7953249 |
| P | Mg | 469.7953249 |
| Cu | Zn | 455.9778154 |
| Fe | Sn | 442.1603058 |
| Cu | S | 442.1603058 |
| P | Se | 428.3427963 |
| Fe | Cr | 428.3427963 |
| Cu | Sb | 428.3427963 |
| Cu | Re | 428.3427963 |
| Cr | Sn | 428.3427963 |
| Fe | Sr | 414.5252867 |
| Fe | Mo | 414.5252867 |
| Fe | Mn | 414.5252867 |
| Sr | Sn | 414.5252867 |
| Cu | Mg | 414.5252867 |
| Mo | Sn | 414.5252867 |
| Sn | Mn | 414.5252867 |
| P | Ca | 400.7077771 |
| Fe | Zn | 400.7077771 |
| Sr | Cr | 400.7077771 |
| Mo | Cr | 400.7077771 |
| Cr | Mn | 400.7077771 |
| Zn | Sn | 400.7077771 |
| P | Ti | 386.8902676 |
| Fe | S | 386.8902676 |
| Sr | Mo | 386.8902676 |
| Sr | Mn | 386.8902676 |
| Mo | Mn | 386.8902676 |
| Cr | Zn | 386.8902676 |
| S | Sn | 386.8902676 |
| P | K | 373.072758 |
| Cu | Se | 373.072758 |
| Fe | Sb | 373.072758 |
| Fe | Re | 373.072758 |
| Sr | Zn | 373.072758 |
| Mo | Zn | 373.072758 |
| Cr | S | 373.072758 |
| Zn | Mn | 373.072758 |
| Sn | Sb | 373.072758 |
| Sn | Re | 373.072758 |
| Fe | Mg | 359.2552485 |
| Sr | S | 359.2552485 |
| Mo | S | 359.2552485 |
| Cr | Sb | 359.2552485 |
| Cr | Re | 359.2552485 |
| S | Mn | 359.2552485 |
| Sn | Mg | 359.2552485 |
| Cu | Ca | 345.4377389 |
| Cr | Mg | 345.4377389 |
| Sr | Sb | 345.4377389 |
| Sr | Re | 345.4377389 |
| Mo | Sb | 345.4377389 |

* Exact permutation *P*<0.01 were showed only.
