# Supplementary material for: Associations between Ionomic Profile and Metabolic Abnormalities in Human Population
Source: PLoS One. 2012 Jun 13;7(6):e38845. doi: 10.1371/journal.pone.0038845 (PMC3374762; doi:10.1371/journal.pone.0038845)
Supplement: Table S11 — The highly connected two ion and three ion modules. (DOC) [file pone.0038845.s011.doc]

**Table S11 The highly connected two ion and three ion modules**

| **The highly connected two ion modules in overweight/obesity** | |  | **The highly connected three ion modules in overweight/obesity** | |
| --- | --- | --- | --- | --- |
| **Ion module** | **score** |  | **Ion module** | **score** |
| Cu_P | 428.3427963 |  | Cu_Mo_P | 248.715172 |
| Cu_Mo | 317.8027198 |  | Cu_P_Sb | 248.715172 |
| Cu_Sb | 303.9852102 |  | Cr_Cu_P | 221.0801529 |
| Mo_P | 303.9852102 |  | Cu_P_Sn | 193.4451338 |
| Cr_Cu | 276.3501911 |  | Cr_Cu_Mo | 179.6276242 |
| P_Sb | 276.3501911 |  | Cr_Mo_P | 179.6276242 |
| Cr_P | 248.715172 |  | Cu_Mg_Mo | 179.6276242 |
| Cu_Sn | 234.8976625 |  | Cu_Mo_Sb | 165.8101147 |
| Mg_Mo | 221.0801529 |  | Mg_Mo_P | 165.8101147 |
| P_Sn | 221.0801529 |  | Mo_P_Sb | 151.9926051 |
| Cr_Mo | 207.2626433 |  | Cr_Cu_Sn | 138.1750956 |
| Mo_Sb | 193.4451338 |  | Cr_P_Sn | 138.1750956 |
| Cu_Mg | 179.6276242 |  | Cu_Mg_P | 138.1750956 |
| Cu_Re | 179.6276242 |  | Cu_P_Re | 138.1750956 |
| Mg_P | 165.8101147 |  | Cu_Sb_Sn | 138.1750956 |
| Cr_Sb | 151.9926051 |  | P_Sb_Sn | 138.1750956 |
| Cr_Sn | 151.9926051 |  | Cr_Cu_Sb | 124.357586 |
| Cu_Mn | 151.9926051 |  | Cr_Mg_Mo | 124.357586 |
| Mo_Sn | 151.9926051 |  | Cr_Mo_Sb | 124.357586 |
| P_Re | 151.9926051 |  | Cr_P_Sb | 124.357586 |
| Sb_Sn | 151.9926051 |  | Cu_Mo_Sn | 124.357586 |
| Fe_P | 138.1750956 |  | Cu_P_Zn | 124.357586 |
| Mo_S | 138.1750956 |  | Cu_Re_Sb | 124.357586 |
| P_Zn | 138.1750956 |  | Mg_Mo_S | 124.357586 |
| Cr_Mg | 124.357586 |  | Cr_Cu_Mg | 110.5400765 |
| Cu_S | 124.357586 |  | Cu_Mo_S | 110.5400765 |
| Cu_Zn | 124.357586 |  | Cu_P_S | 110.5400765 |
| Mg_S | 124.357586 |  | Mo_P_S | 110.5400765 |
| P_S | 124.357586 |  | Mo_P_Sn | 110.5400765 |
| Re_Sb | 124.357586 |  | Cr_Cu_Re | 96.7225669 |
| Cr_S | 110.5400765 |  | Cr_Cu_S | 96.7225669 |
| Cu_Sr | 110.5400765 |  | Cr_Mg_P | 96.7225669 |
| Mn_P | 110.5400765 |  | Cr_Mo_S | 96.7225669 |
| Cr_Re | 96.7225669 |  | Cr_Mo_Sn | 96.7225669 |
| Cu_Fe | 96.7225669 |  | Cr_Sb_Sn | 96.7225669 |
| Fe_Mo | 96.7225669 |  | Cu_Mg_S | 96.7225669 |
| Mg_Sb | 96.7225669 |  | Cu_Mn_P | 96.7225669 |
| Mo_Zn | 96.7225669 |  | Fe_Mo_P | 96.7225669 |
| Cr_Mn | 82.90505734 |  | Mg_Mo_Sb | 96.7225669 |
| Mg_Zn | 82.90505734 |  | Mg_P_S | 96.7225669 |
| Mn_Mo | 82.90505734 |  | P_Re_Sb | 96.7225669 |
| Mo_Re | 82.90505734 |  | Cr_Cu_Mn | 82.90505734 |
| Re_Sn | 82.90505734 |  | Cr_Mg_S | 82.90505734 |
| Sb_Zn | 82.90505734 |  | Cr_P_S | 82.90505734 |
| Cr_Fe | 69.08754778 |  | Cu_Fe_P | 82.90505734 |
| Fe_S | 69.08754778 |  | Cu_Mg_Sb | 82.90505734 |
| Fe_Sb | 69.08754778 |  | Cu_Mn_Mo | 82.90505734 |
| Fe_Zn | 69.08754778 |  | Cu_Mo_Re | 82.90505734 |
| P_Sr | 69.08754778 |  | Cu_Mo_Zn | 82.90505734 |
| S_Zn | 69.08754778 |  | Cu_Re_Sn | 82.90505734 |
| Sb_Sr | 69.08754778 |  | Cu_Sb_Zn | 82.90505734 |
| Ca_P | 55.27003823 |  | Mg_Mo_Zn | 82.90505734 |
| Ca_Sb | 55.27003823 |  | Mo_P_Zn | 82.90505734 |
| Fe_Mg | 55.27003823 |  | Mo_Sb_Sn | 82.90505734 |
| Fe_Mn | 55.27003823 |  | P_Re_Sn | 82.90505734 |
| Fe_Re | 55.27003823 |  | P_Sb_Zn | 82.90505734 |
| Mg_Re | 55.27003823 |  | Cr_P_Re | 69.08754778 |
| Mn_Re | 55.27003823 |  | Cu_Fe_Mo | 69.08754778 |
| Mn_Sb | 55.27003823 |  | Cu_Mg_Zn | 69.08754778 |
| Mn_Sn | 55.27003823 |  | Cu_P_Sr | 69.08754778 |
| Se_Zn | 55.27003823 |  | Cu_Sb_Sr | 69.08754778 |
| Ca_Cu | 41.45252867 |  | Fe_P_Sb | 69.08754778 |
| Ca_Sr | 41.45252867 |  | Mg_P_Zn | 69.08754778 |
| Cr_Zn | 41.45252867 |  | Mo_P_Re | 69.08754778 |
| Cu_Se | 41.45252867 |  | Mo_Re_Sb | 69.08754778 |
| Cu_Ti | 41.45252867 |  | Ca_P_Sb | 55.27003823 |
| Mg_Sn | 41.45252867 |  | Cr_Mg_Sb | 55.27003823 |
| Mn_Sr | 41.45252867 |  | Cr_Mn_P | 55.27003823 |
| Mo_Sr | 41.45252867 |  | Cr_Mo_Re | 55.27003823 |
| P_Se | 41.45252867 |  | Cr_Re_Sb | 55.27003823 |
| Re_S | 41.45252867 |  | Cr_Re_Sn | 55.27003823 |
| S_Sb | 41.45252867 |  | Cu_Fe_Mg | 55.27003823 |
| Sb_Se | 41.45252867 |  | Cu_Fe_Sb | 55.27003823 |
| Sb_Ti | 41.45252867 |  | Cu_Mg_Re | 55.27003823 |
| Se_Ti | 41.45252867 |  | Cu_Mn_Sb | 55.27003823 |
| Sn_Sr | 41.45252867 |  | Cu_Mn_Sn | 55.27003823 |
| Fe_Sn | 27.63501911 |  | Fe_Mg_Mo | 55.27003823 |
| Mg_Mn | 27.63501911 |  | Fe_Mg_P | 55.27003823 |
| Mg_Se | 27.63501911 |  | Fe_Mo_Sb | 55.27003823 |
| Mo_Se | 27.63501911 |  | Mg_Mo_Re | 55.27003823 |
| P_Ti | 27.63501911 |  | Mg_P_Sb | 55.27003823 |
| Re_Sr | 27.63501911 |  | Mn_Mo_P | 55.27003823 |
| Re_Zn | 27.63501911 |  | P_Sb_Sr | 55.27003823 |
| S_Sn | 27.63501911 |  | Ca_Cu_P | 41.45252867 |
| Se_Sn | 27.63501911 |  | Ca_Cu_Sb | 41.45252867 |
| Sn_Zn | 27.63501911 |  | Ca_Cu_Sr | 41.45252867 |
| Ti_Zn | 27.63501911 |  | Ca_P_Sr | 41.45252867 |
| Ca_Cr | 13.81750956 |  | Ca_Sb_Sr | 41.45252867 |
| Ca_Mg | 13.81750956 |  | Cr_Cu_Fe | 41.45252867 |
| Ca_Mn | 13.81750956 |  | Cr_Fe_Mo | 41.45252867 |
| Ca_Mo | 13.81750956 |  | Cr_Fe_P | 41.45252867 |
| Ca_Re | 13.81750956 |  | Cr_Fe_S | 41.45252867 |
| Ca_Zn | 13.81750956 |  | Cr_Mg_Re | 41.45252867 |
| Cr_Sr | 13.81750956 |  | Cr_Mg_Sn | 41.45252867 |
| Fe_Se | 13.81750956 |  | Cr_Mn_Mo | 41.45252867 |
| Fe_Sr | 13.81750956 |  | Cu_Fe_Mn | 41.45252867 |
| Fe_Ti | 13.81750956 |  | Cu_Fe_Re | 41.45252867 |
| K_Mg | 13.81750956 |  | Cu_Fe_S | 41.45252867 |
| K_Mo | 13.81750956 |  | Cu_Mg_Sn | 41.45252867 |
| K_S | 13.81750956 |  | Cu_Mn_Re | 41.45252867 |
| K_Se | 13.81750956 |  | Cu_Mn_Sr | 41.45252867 |
| K_Zn | 13.81750956 |  | Cu_Mo_Sr | 41.45252867 |
| Mg_Sr | 13.81750956 |  | Cu_Re_S | 41.45252867 |
| Mg_Ti | 13.81750956 |  | Cu_S_Sb | 41.45252867 |
| Mn_S | 13.81750956 |  | Cu_Sb_Se | 41.45252867 |
| Mn_Se | 13.81750956 |  | Cu_Sb_Ti | 41.45252867 |
| Mn_Ti | 13.81750956 |  | Cu_Sn_Sr | 41.45252867 |
| Mo_Ti | 13.81750956 |  | Fe_Mn_P | 41.45252867 |
| Re_Ti | 13.81750956 |  | Fe_P_Re | 41.45252867 |
| S_Se | 13.81750956 |  | Fe_P_S | 41.45252867 |
| S_Sr | 13.81750956 |  | Fe_P_Zn | 41.45252867 |
| Se_Sr | 13.81750956 |  | Mg_Mo_Sn | 41.45252867 |
| Sn_Ti | 13.81750956 |  | Mg_P_Re | 41.45252867 |
| Sr_Ti | 13.81750956 |  | Mg_Re_Sb | 41.45252867 |
| Sr_Zn | 13.81750956 |  | Mg_S_Zn | 41.45252867 |
|  |  |  | Mg_Sb_Zn | 41.45252867 |
|  |  |  | Mn_Mo_Sb | 41.45252867 |
|  |  |  | Mn_P_Re | 41.45252867 |
|  |  |  | Mn_P_Sb | 41.45252867 |
|  |  |  | Mn_P_Sn | 41.45252867 |
|  |  |  | Mo_Re_Sn | 41.45252867 |
|  |  |  | Mo_S_Zn | 41.45252867 |
|  |  |  | Mo_Sb_Zn | 41.45252867 |
|  |  |  | P_Se_Zn | 41.45252867 |
|  |  |  | Re_Sb_Sn | 41.45252867 |
|  |  |  | Cr_Cu_Zn | 27.63501911 |
|  |  |  | Cr_Fe_Mg | 27.63501911 |
|  |  |  | Cr_Fe_Mn | 27.63501911 |
|  |  |  | Cr_Mg_Zn | 27.63501911 |
|  |  |  | Cr_Mn_Re | 27.63501911 |
|  |  |  | Cr_Mn_Sn | 27.63501911 |
|  |  |  | Cr_Mo_Zn | 27.63501911 |
|  |  |  | Cr_P_Zn | 27.63501911 |
|  |  |  | Cr_Re_S | 27.63501911 |
|  |  |  | Cr_S_Sb | 27.63501911 |
|  |  |  | Cr_S_Sn | 27.63501911 |
|  |  |  | Cr_S_Zn | 27.63501911 |
|  |  |  | Cu_Fe_Zn | 27.63501911 |
|  |  |  | Cu_Mg_Mn | 27.63501911 |
|  |  |  | Cu_P_Se | 27.63501911 |
|  |  |  | Cu_Re_Sr | 27.63501911 |
|  |  |  | Cu_Re_Zn | 27.63501911 |
|  |  |  | Cu_S_Sn | 27.63501911 |
|  |  |  | Cu_S_Zn | 27.63501911 |
|  |  |  | Cu_Se_Sn | 27.63501911 |
|  |  |  | Cu_Se_Ti | 27.63501911 |
|  |  |  | Cu_Se_Zn | 27.63501911 |
|  |  |  | Cu_Sn_Zn | 27.63501911 |
|  |  |  | Fe_Mg_S | 27.63501911 |
|  |  |  | Fe_Mg_Sb | 27.63501911 |
|  |  |  | Fe_Mg_Zn | 27.63501911 |
|  |  |  | Fe_Mn_Mo | 27.63501911 |
|  |  |  | Fe_Mn_Re | 27.63501911 |
|  |  |  | Fe_Mo_S | 27.63501911 |
|  |  |  | Fe_Mo_Sn | 27.63501911 |
|  |  |  | Fe_Mo_Zn | 27.63501911 |
|  |  |  | Fe_P_Sn | 27.63501911 |
|  |  |  | Fe_Re_Sb | 27.63501911 |
|  |  |  | Fe_S_Zn | 27.63501911 |
|  |  |  | Fe_Sb_Zn | 27.63501911 |
|  |  |  | Mg_Mn_Mo | 27.63501911 |
|  |  |  | Mg_Mo_Se | 27.63501911 |
|  |  |  | Mg_P_Sn | 27.63501911 |
|  |  |  | Mg_Re_S | 27.63501911 |
|  |  |  | Mg_Re_Zn | 27.63501911 |
|  |  |  | Mg_S_Sn | 27.63501911 |
|  |  |  | Mn_Mo_Sn | 27.63501911 |
|  |  |  | Mn_Mo_Sr | 27.63501911 |
|  |  |  | Mn_Re_Sn | 27.63501911 |
|  |  |  | Mn_Sb_Sr | 27.63501911 |
|  |  |  | Mo_Re_S | 27.63501911 |
|  |  |  | Mo_Re_Zn | 27.63501911 |
|  |  |  | Mo_S_Sb | 27.63501911 |
|  |  |  | Mo_S_Sn | 27.63501911 |
|  |  |  | Mo_Sn_Sr | 27.63501911 |
|  |  |  | P_Re_S | 27.63501911 |
|  |  |  | P_Re_Sr | 27.63501911 |
|  |  |  | P_Re_Zn | 27.63501911 |
|  |  |  | P_S_Sb | 27.63501911 |
|  |  |  | P_S_Sn | 27.63501911 |
|  |  |  | P_S_Zn | 27.63501911 |
|  |  |  | P_Sb_Se | 27.63501911 |
|  |  |  | P_Se_Sn | 27.63501911 |
|  |  |  | P_Se_Ti | 27.63501911 |
|  |  |  | P_Sn_Zn | 27.63501911 |
|  |  |  | P_Ti_Zn | 27.63501911 |
|  |  |  | Re_S_Sb | 27.63501911 |
|  |  |  | Re_Sb_Sr | 27.63501911 |
|  |  |  | Re_Sb_Zn | 27.63501911 |
|  |  |  | Sb_Se_Sn | 27.63501911 |
|  |  |  | Sb_Se_Ti | 27.63501911 |
|  |  |  | Sb_Se_Zn | 27.63501911 |
|  |  |  | Sb_Sn_Zn | 27.63501911 |
|  |  |  | Se_Sn_Zn | 27.63501911 |
|  |  |  | Se_Ti_Zn | 27.63501911 |
|  |  |  | Ca_Cr_Mg | 13.81750956 |
|  |  |  | Ca_Cr_Mo | 13.81750956 |
|  |  |  | Ca_Cr_P | 13.81750956 |
|  |  |  | Ca_Cr_Sb | 13.81750956 |
|  |  |  | Ca_Cu_Mn | 13.81750956 |
|  |  |  | Ca_Cu_Re | 13.81750956 |
|  |  |  | Ca_Cu_Zn | 13.81750956 |
|  |  |  | Ca_Mg_Mo | 13.81750956 |
|  |  |  | Ca_Mg_P | 13.81750956 |
|  |  |  | Ca_Mg_Sb | 13.81750956 |
|  |  |  | Ca_Mn_P | 13.81750956 |
|  |  |  | Ca_Mn_Sb | 13.81750956 |
|  |  |  | Ca_Mn_Sr | 13.81750956 |
|  |  |  | Ca_Mo_P | 13.81750956 |
|  |  |  | Ca_Mo_Sb | 13.81750956 |
|  |  |  | Ca_P_Re | 13.81750956 |
|  |  |  | Ca_P_Zn | 13.81750956 |
|  |  |  | Ca_Re_Sb | 13.81750956 |
|  |  |  | Ca_Re_Sr | 13.81750956 |
|  |  |  | Ca_Sb_Zn | 13.81750956 |
|  |  |  | Ca_Sr_Zn | 13.81750956 |
|  |  |  | Cr_Cu_Sr | 13.81750956 |
|  |  |  | Cr_Fe_Re | 13.81750956 |
|  |  |  | Cr_Fe_Sb | 13.81750956 |
|  |  |  | Cr_Fe_Sn | 13.81750956 |
|  |  |  | Cr_Fe_Zn | 13.81750956 |
|  |  |  | Cr_Mg_Mn | 13.81750956 |
|  |  |  | Cr_Mn_S | 13.81750956 |
|  |  |  | Cr_Mn_Sb | 13.81750956 |
|  |  |  | Cr_P_Sr | 13.81750956 |
|  |  |  | Cr_Re_Zn | 13.81750956 |
|  |  |  | Cr_Sb_Zn | 13.81750956 |
|  |  |  | Cr_Sn_Sr | 13.81750956 |
|  |  |  | Cu_Fe_Sr | 13.81750956 |
|  |  |  | Cu_Mg_Se | 13.81750956 |
|  |  |  | Cu_Mg_Sr | 13.81750956 |
|  |  |  | Cu_Mg_Ti | 13.81750956 |
|  |  |  | Cu_Mn_S | 13.81750956 |
|  |  |  | Cu_Mn_Se | 13.81750956 |
|  |  |  | Cu_Mn_Ti | 13.81750956 |
|  |  |  | Cu_Mo_Se | 13.81750956 |
|  |  |  | Cu_Mo_Ti | 13.81750956 |
|  |  |  | Cu_P_Ti | 13.81750956 |
|  |  |  | Cu_Re_Ti | 13.81750956 |
|  |  |  | Cu_S_Sr | 13.81750956 |
|  |  |  | Cu_Se_Sr | 13.81750956 |
|  |  |  | Cu_Sn_Ti | 13.81750956 |
|  |  |  | Cu_Sr_Ti | 13.81750956 |
|  |  |  | Cu_Sr_Zn | 13.81750956 |
|  |  |  | Cu_Ti_Zn | 13.81750956 |
|  |  |  | Fe_Mg_Mn | 13.81750956 |
|  |  |  | Fe_Mg_Re | 13.81750956 |
|  |  |  | Fe_Mn_S | 13.81750956 |
|  |  |  | Fe_Mn_Sb | 13.81750956 |
|  |  |  | Fe_Mo_Re | 13.81750956 |
|  |  |  | Fe_P_Se | 13.81750956 |
|  |  |  | Fe_P_Sr | 13.81750956 |
|  |  |  | Fe_P_Ti | 13.81750956 |
|  |  |  | Fe_Re_S | 13.81750956 |
|  |  |  | Fe_Re_Sr | 13.81750956 |
|  |  |  | Fe_Re_Zn | 13.81750956 |
|  |  |  | Fe_S_Sb | 13.81750956 |
|  |  |  | Fe_S_Sr | 13.81750956 |
|  |  |  | Fe_Sb_Sn | 13.81750956 |
|  |  |  | Fe_Sb_Sr | 13.81750956 |
|  |  |  | Fe_Se_Ti | 13.81750956 |
|  |  |  | Fe_Se_Zn | 13.81750956 |
|  |  |  | Fe_Ti_Zn | 13.81750956 |
|  |  |  | K_Mg_Mo | 13.81750956 |
|  |  |  | K_Mg_S | 13.81750956 |
|  |  |  | K_Mg_Se | 13.81750956 |
|  |  |  | K_Mg_Zn | 13.81750956 |
|  |  |  | K_Mo_S | 13.81750956 |
|  |  |  | K_Mo_Se | 13.81750956 |
|  |  |  | K_Mo_Zn | 13.81750956 |
|  |  |  | K_S_Se | 13.81750956 |
|  |  |  | K_S_Zn | 13.81750956 |
|  |  |  | K_Se_Zn | 13.81750956 |
|  |  |  | Mg_Mn_P | 13.81750956 |
|  |  |  | Mg_Mn_S | 13.81750956 |
|  |  |  | Mg_Mn_Sb | 13.81750956 |
|  |  |  | Mg_Mn_Se | 13.81750956 |
|  |  |  | Mg_Mn_Sr | 13.81750956 |
|  |  |  | Mg_Mn_Ti | 13.81750956 |
|  |  |  | Mg_Mo_Sr | 13.81750956 |
|  |  |  | Mg_Mo_Ti | 13.81750956 |
|  |  |  | Mg_Re_Sn | 13.81750956 |
|  |  |  | Mg_S_Sb | 13.81750956 |
|  |  |  | Mg_S_Se | 13.81750956 |
|  |  |  | Mg_Sb_Se | 13.81750956 |
|  |  |  | Mg_Sb_Sn | 13.81750956 |
|  |  |  | Mg_Sb_Sr | 13.81750956 |
|  |  |  | Mg_Sb_Ti | 13.81750956 |
|  |  |  | Mg_Se_Sr | 13.81750956 |
|  |  |  | Mg_Se_Ti | 13.81750956 |
|  |  |  | Mg_Se_Zn | 13.81750956 |
|  |  |  | Mg_Sr_Ti | 13.81750956 |
|  |  |  | Mn_Mo_S | 13.81750956 |
|  |  |  | Mn_Mo_Se | 13.81750956 |
|  |  |  | Mn_Mo_Ti | 13.81750956 |
|  |  |  | Mn_P_S | 13.81750956 |
|  |  |  | Mn_P_Sr | 13.81750956 |
|  |  |  | Mn_Sb_Se | 13.81750956 |
|  |  |  | Mn_Sb_Sn | 13.81750956 |
|  |  |  | Mn_Sb_Ti | 13.81750956 |
|  |  |  | Mn_Se_Sr | 13.81750956 |
|  |  |  | Mn_Se_Ti | 13.81750956 |
|  |  |  | Mn_Sn_Sr | 13.81750956 |
|  |  |  | Mn_Sr_Ti | 13.81750956 |
|  |  |  | Mo_S_Se | 13.81750956 |
|  |  |  | Mo_Sb_Se | 13.81750956 |
|  |  |  | Mo_Sb_Sr | 13.81750956 |
|  |  |  | Mo_Sb_Ti | 13.81750956 |
|  |  |  | Mo_Se_Sr | 13.81750956 |
|  |  |  | Mo_Se_Ti | 13.81750956 |
|  |  |  | Mo_Se_Zn | 13.81750956 |
|  |  |  | Mo_Sr_Ti | 13.81750956 |
|  |  |  | P_S_Sr | 13.81750956 |
|  |  |  | P_Sb_Ti | 13.81750956 |
|  |  |  | P_Sn_Sr | 13.81750956 |
|  |  |  | P_Sn_Ti | 13.81750956 |
|  |  |  | P_Sr_Zn | 13.81750956 |
|  |  |  | Re_S_Sn | 13.81750956 |
|  |  |  | Re_S_Sr | 13.81750956 |
|  |  |  | Re_Sb_Ti | 13.81750956 |
|  |  |  | S_Sb_Sr | 13.81750956 |
|  |  |  | S_Se_Zn | 13.81750956 |
|  |  |  | Sb_Se_Sr | 13.81750956 |
|  |  |  | Sb_Sn_Ti | 13.81750956 |
|  |  |  | Sb_Sr_Ti | 13.81750956 |
|  |  |  | Sb_Sr_Zn | 13.81750956 |
|  |  |  | Sb_Ti_Zn | 13.81750956 |
|  |  |  | Se_Sn_Ti | 13.81750956 |
|  |  |  | Se_Sr_Ti | 13.81750956 |
|  |  |  | Sn_Ti_Zn | 13.81750956 |

| **The highly connected two ion modules in metabolic syndrome** | |  | **The highly connected three ion modules in metabolic syndrome** | |
| --- | --- | --- | --- | --- |
| **Ion module** | **score** |  | **Ion module** | **score** |
| Cu_P | 317.8027198 |  | Cr_Cu_P | 179.6276242 |
| Mn_P | 303.9852102 |  | Mg_Mo_P | 165.8101147 |
| P_Zn | 262.5326816 |  | Cu_P_Sn | 138.1750956 |
| Mg_P | 248.715172 |  | Mg_Mo_S | 138.1750956 |
| Cr_P | 234.8976625 |  | Mg_P_S | 138.1750956 |
| Mo_P | 234.8976625 |  | Cr_Mg_P | 124.357586 |
| P_Sn | 234.8976625 |  | Cu_P_Sb | 124.357586 |
| Cr_Cu | 221.0801529 |  | Cu_P_Zn | 124.357586 |
| Mg_Mo | 207.2626433 |  | Mg_Mn_P | 124.357586 |
| P_S | 193.4451338 |  | Cr_Cu_Mo | 110.5400765 |
| Cu_Mo | 179.6276242 |  | Cr_Cu_Sb | 110.5400765 |
| P_Sb | 179.6276242 |  | Cr_Mn_P | 110.5400765 |
| Cu_Sn | 165.8101147 |  | Cr_P_Re | 110.5400765 |
| Mg_S | 165.8101147 |  | Cu_Mg_P | 110.5400765 |
| P_Re | 165.8101147 |  | Cu_Mn_P | 110.5400765 |
| P_Sr | 165.8101147 |  | Cu_Mo_P | 110.5400765 |
| Cu_Sb | 151.9926051 |  | Cu_P_Re | 110.5400765 |
| Fe_P | 151.9926051 |  | Cu_P_S | 110.5400765 |
| Mo_Zn | 151.9926051 |  | Mn_Mo_P | 110.5400765 |
| Cr_Mg | 138.1750956 |  | Mo_P_S | 110.5400765 |
| Cr_Mn | 138.1750956 |  | Mo_P_Zn | 110.5400765 |
| Cr_Mo | 138.1750956 |  | Cr_Cu_Mg | 96.7225669 |
| Cr_Re | 138.1750956 |  | Cr_Cu_Mn | 96.7225669 |
| Cu_Zn | 138.1750956 |  | Cr_Cu_Re | 96.7225669 |
| Mo_S | 138.1750956 |  | Cr_Cu_Sn | 96.7225669 |
| S_Zn | 138.1750956 |  | Cr_Mg_S | 96.7225669 |
| Cr_S | 124.357586 |  | Cr_P_S | 96.7225669 |
| Cr_Sb | 124.357586 |  | Cr_P_Sb | 96.7225669 |
| Cu_Mg | 124.357586 |  | Mg_Mn_Mo | 96.7225669 |
| Cu_Mn | 124.357586 |  | Mn_P_Re | 96.7225669 |
| Cu_Re | 124.357586 |  | Mn_P_Sn | 96.7225669 |
| Fe_Zn | 124.357586 |  | Mn_P_Zn | 96.7225669 |
| Mg_Mn | 124.357586 |  | P_Se_Zn | 96.7225669 |
| Mn_Mo | 124.357586 |  | P_Sr_Zn | 96.7225669 |
| Mn_Re | 124.357586 |  | Cr_Mg_Mo | 82.90505734 |
| Mn_Sn | 124.357586 |  | Cr_Mn_Re | 82.90505734 |
| Mn_Zn | 124.357586 |  | Cr_Mo_P | 82.90505734 |
| P_Se | 124.357586 |  | Cu_Mg_Mo | 82.90505734 |
| Se_Zn | 124.357586 |  | Cu_Sb_Sn | 82.90505734 |
| Cr_Zn | 110.5400765 |  | Fe_Mn_P | 82.90505734 |
| Cu_S | 110.5400765 |  | Mg_Mo_Zn | 82.90505734 |
| Fe_Mn | 110.5400765 |  | Mg_P_Zn | 82.90505734 |
| Fe_Mo | 110.5400765 |  | Mn_P_S | 82.90505734 |
| Mg_Zn | 110.5400765 |  | P_S_Zn | 82.90505734 |
| Sb_Sn | 110.5400765 |  | P_Sn_Zn | 82.90505734 |
| Cr_Sn | 96.7225669 |  | Cr_Cu_S | 69.08754778 |
| Mn_Sb | 96.7225669 |  | Cr_Cu_Zn | 69.08754778 |
| Mo_Sb | 96.7225669 |  | Cr_Mo_S | 69.08754778 |
| P_Ti | 96.7225669 |  | Cr_P_Sn | 69.08754778 |
| Re_Zn | 96.7225669 |  | Cr_P_Zn | 69.08754778 |
| Sn_Zn | 96.7225669 |  | Cr_Re_Sb | 69.08754778 |
| Sr_Zn | 96.7225669 |  | Cr_S_Zn | 69.08754778 |
| Fe_Mg | 82.90505734 |  | Cr_Sb_Sn | 69.08754778 |
| Mn_S | 82.90505734 |  | Cu_Mg_S | 69.08754778 |
| Mo_Se | 82.90505734 |  | Cu_Mo_Zn | 69.08754778 |
| Re_Sb | 82.90505734 |  | Cu_P_Sr | 69.08754778 |
| Sb_Sr | 82.90505734 |  | Cu_S_Zn | 69.08754778 |
| Ca_P | 69.08754778 |  | Fe_Mg_P | 69.08754778 |
| Cr_Fe | 69.08754778 |  | Mg_Mn_S | 69.08754778 |
| Cu_Fe | 69.08754778 |  | Mg_P_Sb | 69.08754778 |
| Cu_Sr | 69.08754778 |  | Mg_S_Zn | 69.08754778 |
| Fe_S | 69.08754778 |  | Mn_P_Sb | 69.08754778 |
| Fe_Sb | 69.08754778 |  | Mn_P_Sr | 69.08754778 |
| Fe_Sn | 69.08754778 |  | Mo_Se_Zn | 69.08754778 |
| K_Mn | 69.08754778 |  | P_Re_Sb | 69.08754778 |
| K_P | 69.08754778 |  | P_Re_Sn | 69.08754778 |
| K_Se | 69.08754778 |  | P_Re_Sr | 69.08754778 |
| Mg_Sb | 69.08754778 |  | P_Re_Zn | 69.08754778 |
| Mn_Sr | 69.08754778 |  | P_Sb_Sn | 69.08754778 |
| Mo_Sn | 69.08754778 |  | P_Sb_Sr | 69.08754778 |
| Re_Sn | 69.08754778 |  | Ca_Mn_P | 55.27003823 |
| Re_Sr | 69.08754778 |  | Cr_Cu_Fe | 55.27003823 |
| Sb_Ti | 69.08754778 |  | Cr_Mg_Zn | 55.27003823 |
| Sb_Zn | 69.08754778 |  | Cr_Mn_Mo | 55.27003823 |
| Sn_Ti | 69.08754778 |  | Cr_Mn_Sb | 55.27003823 |
| Ca_Mn | 55.27003823 |  | Cr_Mo_Zn | 55.27003823 |
| Ca_Sb | 55.27003823 |  | Cu_Fe_Mg | 55.27003823 |
| Ca_Zn | 55.27003823 |  | Cu_Fe_Mo | 55.27003823 |
| Cu_Se | 55.27003823 |  | Cu_Mn_Mo | 55.27003823 |
| Fe_Se | 55.27003823 |  | Cu_Mn_Re | 55.27003823 |
| Fe_Ti | 55.27003823 |  | Cu_Mn_Sn | 55.27003823 |
| K_Mg | 55.27003823 |  | Cu_Mo_S | 55.27003823 |
| K_Mo | 55.27003823 |  | Cu_P_Se | 55.27003823 |
| K_Zn | 55.27003823 |  | Cu_Re_Sb | 55.27003823 |
| Mg_Se | 55.27003823 |  | Cu_Re_Sn | 55.27003823 |
| Mg_Sn | 55.27003823 |  | Fe_Mg_Mo | 55.27003823 |
| Mo_Sr | 55.27003823 |  | Fe_Mo_P | 55.27003823 |
| Mo_Ti | 55.27003823 |  | Fe_P_Zn | 55.27003823 |
| Re_S | 55.27003823 |  | K_Mg_Zn | 55.27003823 |
| S_Se | 55.27003823 |  | K_Mn_P | 55.27003823 |
| S_Sn | 55.27003823 |  | K_Mo_Se | 55.27003823 |
| S_Sr | 55.27003823 |  | Mg_Mo_Se | 55.27003823 |
| Sb_Se | 55.27003823 |  | Mg_P_Sn | 55.27003823 |
| Se_Sn | 55.27003823 |  | Mg_Se_Zn | 55.27003823 |
| Se_Ti | 55.27003823 |  | Mn_Mo_S | 55.27003823 |
| Sn_Sr | 55.27003823 |  | Mn_Re_Sb | 55.27003823 |
| Ti_Zn | 55.27003823 |  | Mo_S_Zn | 55.27003823 |
| Ca_Cr | 41.45252867 |  | Mo_Sb_Ti | 55.27003823 |
| Ca_Cu | 41.45252867 |  | P_Re_S | 55.27003823 |
| Ca_Fe | 41.45252867 |  | P_S_Sn | 55.27003823 |
| Ca_Re | 41.45252867 |  | P_S_Sr | 55.27003823 |
| Ca_Sn | 41.45252867 |  | P_Sb_Zn | 55.27003823 |
| Cr_K | 41.45252867 |  | P_Se_Ti | 55.27003823 |
| Cr_Sr | 41.45252867 |  | P_Sn_Sr | 55.27003823 |
| Cu_Ti | 41.45252867 |  | P_Ti_Zn | 55.27003823 |
| Fe_Re | 41.45252867 |  | S_Se_Zn | 55.27003823 |
| K_Re | 41.45252867 |  | Ca_Cr_Re | 41.45252867 |
| K_Sn | 41.45252867 |  | Ca_P_Sb | 41.45252867 |
| Mg_Re | 41.45252867 |  | Ca_P_Zn | 41.45252867 |
| Mg_Sr | 41.45252867 |  | Cr_Fe_Mg | 41.45252867 |
| Mn_Se | 41.45252867 |  | Cr_Fe_Mo | 41.45252867 |
| Mn_Ti | 41.45252867 |  | Cr_Fe_P | 41.45252867 |
| S_Sb | 41.45252867 |  | Cr_Fe_S | 41.45252867 |
| Sr_Ti | 41.45252867 |  | Cr_Fe_Zn | 41.45252867 |
| Ca_Mo | 27.63501911 |  | Cr_Mg_Mn | 41.45252867 |
| Cr_Se | 27.63501911 |  | Cr_Mg_Sb | 41.45252867 |
| Cr_Ti | 27.63501911 |  | Cr_Mn_S | 41.45252867 |
| Cu_K | 27.63501911 |  | Cr_P_Sr | 41.45252867 |
| Fe_K | 27.63501911 |  | Cr_Re_Sn | 41.45252867 |
| Fe_Sr | 27.63501911 |  | Cr_Re_Sr | 41.45252867 |
| K_S | 27.63501911 |  | Cr_Re_Zn | 41.45252867 |
| K_Sb | 27.63501911 |  | Cr_Sb_Sr | 41.45252867 |
| K_Sr | 27.63501911 |  | Cu_Fe_P | 41.45252867 |
| K_Ti | 27.63501911 |  | Cu_Mg_Sb | 41.45252867 |
| Mo_Re | 27.63501911 |  | Cu_Mg_Zn | 41.45252867 |
| Re_Se | 27.63501911 |  | Cu_Mn_Sb | 41.45252867 |
| Re_Ti | 27.63501911 |  | Cu_Mo_Sb | 41.45252867 |
| Ca_K | 13.81750956 |  | Cu_Re_Zn | 41.45252867 |
| Ca_Se | 13.81750956 |  | Cu_S_Sn | 41.45252867 |
| Ca_Sr | 13.81750956 |  | Cu_Sb_Sr | 41.45252867 |
| Mg_Ti | 13.81750956 |  | Cu_Sb_Ti | 41.45252867 |
| S_Ti | 13.81750956 |  | Cu_Se_Sn | 41.45252867 |
| Se_Sr | 13.81750956 |  | Cu_Se_Zn | 41.45252867 |
|  |  |  | Cu_Sn_Ti | 41.45252867 |
|  |  |  | Cu_Sn_Zn | 41.45252867 |
|  |  |  | Cu_Sr_Zn | 41.45252867 |
|  |  |  | Fe_Mg_Mn | 41.45252867 |
|  |  |  | Fe_Mg_S | 41.45252867 |
|  |  |  | Fe_Mn_Sn | 41.45252867 |
|  |  |  | Fe_Mn_Zn | 41.45252867 |
|  |  |  | Fe_Mo_Sb | 41.45252867 |
|  |  |  | Fe_P_S | 41.45252867 |
|  |  |  | Fe_P_Se | 41.45252867 |
|  |  |  | Fe_P_Sn | 41.45252867 |
|  |  |  | Fe_P_Ti | 41.45252867 |
|  |  |  | Fe_S_Zn | 41.45252867 |
|  |  |  | Fe_Sb_Zn | 41.45252867 |
|  |  |  | Fe_Se_Ti | 41.45252867 |
|  |  |  | Fe_Sn_Zn | 41.45252867 |
|  |  |  | K_Mg_Mo | 41.45252867 |
|  |  |  | K_Mg_Se | 41.45252867 |
|  |  |  | K_Mo_Zn | 41.45252867 |
|  |  |  | K_Se_Zn | 41.45252867 |
|  |  |  | Mg_Mn_Zn | 41.45252867 |
|  |  |  | Mg_P_Re | 41.45252867 |
|  |  |  | Mg_P_Sr | 41.45252867 |
|  |  |  | Mn_Mo_Zn | 41.45252867 |
|  |  |  | Mn_P_Se | 41.45252867 |
|  |  |  | Mn_Re_Sr | 41.45252867 |
|  |  |  | Mn_Re_Zn | 41.45252867 |
|  |  |  | Mn_Sb_Sr | 41.45252867 |
|  |  |  | Mo_P_Sb | 41.45252867 |
|  |  |  | Mo_P_Se | 41.45252867 |
|  |  |  | Mo_P_Sr | 41.45252867 |
|  |  |  | Mo_Sb_Sn | 41.45252867 |
|  |  |  | P_S_Sb | 41.45252867 |
|  |  |  | P_Sb_Se | 41.45252867 |
|  |  |  | P_Se_Sn | 41.45252867 |
|  |  |  | P_Sn_Ti | 41.45252867 |
|  |  |  | Re_S_Sr | 41.45252867 |
|  |  |  | Re_Sb_Sr | 41.45252867 |
|  |  |  | Sb_Se_Sn | 41.45252867 |
|  |  |  | Sb_Sn_Ti | 41.45252867 |
|  |  |  | Sn_Sr_Zn | 41.45252867 |
|  |  |  | Ca_Cr_Cu | 27.63501911 |
|  |  |  | Ca_Cr_Mn | 27.63501911 |
|  |  |  | Ca_Cr_P | 27.63501911 |
|  |  |  | Ca_Cr_Zn | 27.63501911 |
|  |  |  | Ca_Cu_P | 27.63501911 |
|  |  |  | Ca_Cu_Re | 27.63501911 |
|  |  |  | Ca_Cu_Sb | 27.63501911 |
|  |  |  | Ca_Cu_Zn | 27.63501911 |
|  |  |  | Ca_Fe_Mo | 27.63501911 |
|  |  |  | Ca_Fe_Sb | 27.63501911 |
|  |  |  | Ca_Fe_Sn | 27.63501911 |
|  |  |  | Ca_Fe_Zn | 27.63501911 |
|  |  |  | Ca_Mn_Re | 27.63501911 |
|  |  |  | Ca_Mn_Sb | 27.63501911 |
|  |  |  | Ca_Mn_Sn | 27.63501911 |
|  |  |  | Ca_Mn_Zn | 27.63501911 |
|  |  |  | Ca_P_Re | 27.63501911 |
|  |  |  | Ca_P_Sn | 27.63501911 |
|  |  |  | Ca_Re_Zn | 27.63501911 |
|  |  |  | Ca_Sb_Sn | 27.63501911 |
|  |  |  | Ca_Sb_Zn | 27.63501911 |
|  |  |  | Cr_Cu_Sr | 27.63501911 |
|  |  |  | Cr_Cu_Ti | 27.63501911 |
|  |  |  | Cr_Fe_Re | 27.63501911 |
|  |  |  | Cr_K_Mn | 27.63501911 |
|  |  |  | Cr_K_Re | 27.63501911 |
|  |  |  | Cr_Mg_Re | 27.63501911 |
|  |  |  | Cr_Mg_Sn | 27.63501911 |
|  |  |  | Cr_Mn_Sn | 27.63501911 |
|  |  |  | Cr_Mn_Sr | 27.63501911 |
|  |  |  | Cr_Mn_Zn | 27.63501911 |
|  |  |  | Cr_Mo_Re | 27.63501911 |
|  |  |  | Cr_Mo_Sb | 27.63501911 |
|  |  |  | Cr_Mo_Sn | 27.63501911 |
|  |  |  | Cr_Mo_Ti | 27.63501911 |
|  |  |  | Cr_Re_S | 27.63501911 |
|  |  |  | Cr_S_Sb | 27.63501911 |
|  |  |  | Cr_S_Sn | 27.63501911 |
|  |  |  | Cr_S_Sr | 27.63501911 |
|  |  |  | Cr_Sb_Ti | 27.63501911 |
|  |  |  | Cr_Se_Zn | 27.63501911 |
|  |  |  | Cr_Sn_Ti | 27.63501911 |
|  |  |  | Cr_Sn_Zn | 27.63501911 |
|  |  |  | Cu_Fe_Re | 27.63501911 |
|  |  |  | Cu_Fe_S | 27.63501911 |
|  |  |  | Cu_Fe_Zn | 27.63501911 |
|  |  |  | Cu_K_Mn | 27.63501911 |
|  |  |  | Cu_K_P | 27.63501911 |
|  |  |  | Cu_Mg_Mn | 27.63501911 |
|  |  |  | Cu_Mg_Sn | 27.63501911 |
|  |  |  | Cu_Mn_S | 27.63501911 |
|  |  |  | Cu_Mo_Re | 27.63501911 |
|  |  |  | Cu_Mo_Sn | 27.63501911 |
|  |  |  | Cu_Mo_Ti | 27.63501911 |
|  |  |  | Cu_Re_S | 27.63501911 |
|  |  |  | Cu_Re_Se | 27.63501911 |
|  |  |  | Cu_Re_Sr | 27.63501911 |
|  |  |  | Cu_S_Sb | 27.63501911 |
|  |  |  | Cu_S_Se | 27.63501911 |
|  |  |  | Cu_S_Sr | 27.63501911 |
|  |  |  | Cu_Sb_Se | 27.63501911 |
|  |  |  | Cu_Sb_Zn | 27.63501911 |
|  |  |  | Cu_Sn_Sr | 27.63501911 |
|  |  |  | Fe_K_Se | 27.63501911 |
|  |  |  | Fe_Mn_Mo | 27.63501911 |
|  |  |  | Fe_Mn_S | 27.63501911 |
|  |  |  | Fe_Mn_Sb | 27.63501911 |
|  |  |  | Fe_Mo_Re | 27.63501911 |
|  |  |  | Fe_Mo_S | 27.63501911 |
|  |  |  | Fe_Mo_Se | 27.63501911 |
|  |  |  | Fe_Mo_Ti | 27.63501911 |
|  |  |  | Fe_Mo_Zn | 27.63501911 |
|  |  |  | Fe_P_Sb | 27.63501911 |
|  |  |  | Fe_Re_Zn | 27.63501911 |
|  |  |  | Fe_Sb_Se | 27.63501911 |
|  |  |  | Fe_Sb_Sn | 27.63501911 |
|  |  |  | Fe_Sb_Ti | 27.63501911 |
|  |  |  | Fe_Se_Zn | 27.63501911 |
|  |  |  | Fe_Ti_Zn | 27.63501911 |
|  |  |  | K_Mg_P | 27.63501911 |
|  |  |  | K_Mg_S | 27.63501911 |
|  |  |  | K_Mn_Re | 27.63501911 |
|  |  |  | K_Mn_Se | 27.63501911 |
|  |  |  | K_Mo_S | 27.63501911 |
|  |  |  | K_P_Re | 27.63501911 |
|  |  |  | K_P_Se | 27.63501911 |
|  |  |  | K_P_Sn | 27.63501911 |
|  |  |  | K_P_Sr | 27.63501911 |
|  |  |  | K_P_Ti | 27.63501911 |
|  |  |  | K_P_Zn | 27.63501911 |
|  |  |  | K_Re_Sr | 27.63501911 |
|  |  |  | K_S_Se | 27.63501911 |
|  |  |  | K_S_Zn | 27.63501911 |
|  |  |  | Mg_Mn_Sb | 27.63501911 |
|  |  |  | Mg_Mn_Se | 27.63501911 |
|  |  |  | Mg_Mn_Sr | 27.63501911 |
|  |  |  | Mg_Mo_Sb | 27.63501911 |
|  |  |  | Mg_P_Se | 27.63501911 |
|  |  |  | Mg_Re_Sr | 27.63501911 |
|  |  |  | Mg_S_Sb | 27.63501911 |
|  |  |  | Mg_S_Se | 27.63501911 |
|  |  |  | Mg_S_Sn | 27.63501911 |
|  |  |  | Mg_Sb_Sr | 27.63501911 |
|  |  |  | Mg_Sn_Zn | 27.63501911 |
|  |  |  | Mn_Mo_Sb | 27.63501911 |
|  |  |  | Mn_Mo_Se | 27.63501911 |
|  |  |  | Mn_Mo_Sn | 27.63501911 |
|  |  |  | Mn_P_Ti | 27.63501911 |
|  |  |  | Mn_Re_S | 27.63501911 |
|  |  |  | Mn_Re_Sn | 27.63501911 |
|  |  |  | Mn_S_Sr | 27.63501911 |
|  |  |  | Mn_S_Zn | 27.63501911 |
|  |  |  | Mn_Sb_Sn | 27.63501911 |
|  |  |  | Mn_Sb_Zn | 27.63501911 |
|  |  |  | Mn_Se_Zn | 27.63501911 |
|  |  |  | Mn_Sn_Zn | 27.63501911 |
|  |  |  | Mn_Sr_Zn | 27.63501911 |
|  |  |  | Mo_P_Sn | 27.63501911 |
|  |  |  | Mo_S_Se | 27.63501911 |
|  |  |  | Mo_Sb_Se | 27.63501911 |
|  |  |  | Mo_Sb_Sr | 27.63501911 |
|  |  |  | Mo_Sn_Ti | 27.63501911 |
|  |  |  | Mo_Sr_Zn | 27.63501911 |
|  |  |  | P_Re_Se | 27.63501911 |
|  |  |  | P_Re_Ti | 27.63501911 |
|  |  |  | P_S_Se | 27.63501911 |
|  |  |  | P_Sb_Ti | 27.63501911 |
|  |  |  | P_Sr_Ti | 27.63501911 |
|  |  |  | Re_S_Sb | 27.63501911 |
|  |  |  | Re_S_Zn | 27.63501911 |
|  |  |  | Re_Sb_Sn | 27.63501911 |
|  |  |  | Re_Sb_Zn | 27.63501911 |
|  |  |  | Re_Se_Zn | 27.63501911 |
|  |  |  | Re_Sn_Sr | 27.63501911 |
|  |  |  | Re_Sn_Zn | 27.63501911 |
|  |  |  | Re_Sr_Ti | 27.63501911 |
|  |  |  | Re_Sr_Zn | 27.63501911 |
|  |  |  | Re_Ti_Zn | 27.63501911 |
|  |  |  | S_Sb_Sr | 27.63501911 |
|  |  |  | S_Sn_Sr | 27.63501911 |
|  |  |  | S_Sn_Zn | 27.63501911 |
|  |  |  | S_Sr_Zn | 27.63501911 |
|  |  |  | Sb_Se_Ti | 27.63501911 |
|  |  |  | Sb_Se_Zn | 27.63501911 |
|  |  |  | Sb_Sn_Zn | 27.63501911 |
|  |  |  | Se_Sn_Zn | 27.63501911 |
|  |  |  | Se_Ti_Zn | 27.63501911 |
|  |  |  | Sr_Ti_Zn | 27.63501911 |
|  |  |  | Ca_Cr_Fe | 13.81750956 |
|  |  |  | Ca_Cr_Mo | 13.81750956 |
|  |  |  | Ca_Cr_Sb | 13.81750956 |
|  |  |  | Ca_Cu_Fe | 13.81750956 |
|  |  |  | Ca_Cu_Mn | 13.81750956 |
|  |  |  | Ca_Cu_Mo | 13.81750956 |
|  |  |  | Ca_Cu_Sr | 13.81750956 |
|  |  |  | Ca_Fe_K | 13.81750956 |
|  |  |  | Ca_Fe_Mn | 13.81750956 |
|  |  |  | Ca_Fe_P | 13.81750956 |
|  |  |  | Ca_Fe_Re | 13.81750956 |
|  |  |  | Ca_Fe_Se | 13.81750956 |
|  |  |  | Ca_K_Mo | 13.81750956 |
|  |  |  | Ca_K_Sb | 13.81750956 |
|  |  |  | Ca_K_Se | 13.81750956 |
|  |  |  | Ca_K_Sn | 13.81750956 |
|  |  |  | Ca_Mo_Re | 13.81750956 |
|  |  |  | Ca_Mo_Sb | 13.81750956 |
|  |  |  | Ca_Mo_Se | 13.81750956 |
|  |  |  | Ca_Mo_Sn | 13.81750956 |
|  |  |  | Ca_Mo_Zn | 13.81750956 |
|  |  |  | Ca_P_Sr | 13.81750956 |
|  |  |  | Ca_Re_Sb | 13.81750956 |
|  |  |  | Ca_Sb_Se | 13.81750956 |
|  |  |  | Ca_Sb_Sr | 13.81750956 |
|  |  |  | Ca_Se_Sn | 13.81750956 |
|  |  |  | Ca_Sn_Zn | 13.81750956 |
|  |  |  | Ca_Sr_Zn | 13.81750956 |
|  |  |  | Cr_Cu_K | 13.81750956 |
|  |  |  | Cr_Cu_Se | 13.81750956 |
|  |  |  | Cr_Fe_Mn | 13.81750956 |
|  |  |  | Cr_Fe_Sn | 13.81750956 |
|  |  |  | Cr_K_Mg | 13.81750956 |
|  |  |  | Cr_K_Mo | 13.81750956 |
|  |  |  | Cr_K_P | 13.81750956 |
|  |  |  | Cr_K_S | 13.81750956 |
|  |  |  | Cr_K_Sb | 13.81750956 |
|  |  |  | Cr_K_Se | 13.81750956 |
|  |  |  | Cr_K_Sr | 13.81750956 |
|  |  |  | Cr_K_Zn | 13.81750956 |
|  |  |  | Cr_Mg_Se | 13.81750956 |
|  |  |  | Cr_Mg_Sr | 13.81750956 |
|  |  |  | Cr_Mn_Ti | 13.81750956 |
|  |  |  | Cr_Mo_Se | 13.81750956 |
|  |  |  | Cr_P_Se | 13.81750956 |
|  |  |  | Cr_Re_Se | 13.81750956 |
|  |  |  | Cr_S_Se | 13.81750956 |
|  |  |  | Cr_Sb_Se | 13.81750956 |
|  |  |  | Cr_Sb_Zn | 13.81750956 |
|  |  |  | Cr_Se_Sn | 13.81750956 |
|  |  |  | Cr_Sn_Sr | 13.81750956 |
|  |  |  | Cu_Fe_Mn | 13.81750956 |
|  |  |  | Cu_Fe_Sn | 13.81750956 |
|  |  |  | Cu_K_Re | 13.81750956 |
|  |  |  | Cu_K_Sb | 13.81750956 |
|  |  |  | Cu_K_Sn | 13.81750956 |
|  |  |  | Cu_K_Sr | 13.81750956 |
|  |  |  | Cu_Mg_Re | 13.81750956 |
|  |  |  | Cu_Mn_Sr | 13.81750956 |
|  |  |  | Cu_Mn_Ti | 13.81750956 |
|  |  |  | Cu_Mn_Zn | 13.81750956 |
|  |  |  | Cu_Mo_Sr | 13.81750956 |
|  |  |  | Cu_P_Ti | 13.81750956 |
|  |  |  | Cu_Se_Sr | 13.81750956 |
|  |  |  | Cu_Se_Ti | 13.81750956 |
|  |  |  | Fe_K_Mn | 13.81750956 |
|  |  |  | Fe_K_Mo | 13.81750956 |
|  |  |  | Fe_K_P | 13.81750956 |
|  |  |  | Fe_K_Sb | 13.81750956 |
|  |  |  | Fe_K_Sn | 13.81750956 |
|  |  |  | Fe_K_Ti | 13.81750956 |
|  |  |  | Fe_Mg_Re | 13.81750956 |
|  |  |  | Fe_Mg_Sn | 13.81750956 |
|  |  |  | Fe_Mg_Zn | 13.81750956 |
|  |  |  | Fe_Mn_Re | 13.81750956 |
|  |  |  | Fe_Mn_Se | 13.81750956 |
|  |  |  | Fe_Mn_Ti | 13.81750956 |
|  |  |  | Fe_Mo_Sn | 13.81750956 |
|  |  |  | Fe_Mo_Sr | 13.81750956 |
|  |  |  | Fe_P_Re | 13.81750956 |
|  |  |  | Fe_P_Sr | 13.81750956 |
|  |  |  | Fe_Re_Sb | 13.81750956 |
|  |  |  | Fe_S_Sn | 13.81750956 |
|  |  |  | Fe_Sb_Sr | 13.81750956 |
|  |  |  | Fe_Se_Sn | 13.81750956 |
|  |  |  | Fe_Sr_Ti | 13.81750956 |
|  |  |  | K_Mg_Mn | 13.81750956 |
|  |  |  | K_Mg_Re | 13.81750956 |
|  |  |  | K_Mg_Sn | 13.81750956 |
|  |  |  | K_Mg_Sr | 13.81750956 |
|  |  |  | K_Mg_Ti | 13.81750956 |
|  |  |  | K_Mn_Mo | 13.81750956 |
|  |  |  | K_Mn_Sb | 13.81750956 |
|  |  |  | K_Mn_Sn | 13.81750956 |
|  |  |  | K_Mn_Sr | 13.81750956 |
|  |  |  | K_Mn_Ti | 13.81750956 |
|  |  |  | K_Mn_Zn | 13.81750956 |
|  |  |  | K_Mo_P | 13.81750956 |
|  |  |  | K_Mo_Sb | 13.81750956 |
|  |  |  | K_Mo_Sn | 13.81750956 |
|  |  |  | K_P_Sb | 13.81750956 |
|  |  |  | K_Re_Sb | 13.81750956 |
|  |  |  | K_Re_Sn | 13.81750956 |
|  |  |  | K_Re_Ti | 13.81750956 |
|  |  |  | K_Re_Zn | 13.81750956 |
|  |  |  | K_Sb_Se | 13.81750956 |
|  |  |  | K_Sb_Sn | 13.81750956 |
|  |  |  | K_Sb_Sr | 13.81750956 |
|  |  |  | K_Se_Sn | 13.81750956 |
|  |  |  | K_Se_Ti | 13.81750956 |
|  |  |  | K_Sn_Sr | 13.81750956 |
|  |  |  | K_Sn_Ti | 13.81750956 |
|  |  |  | K_Sn_Zn | 13.81750956 |
|  |  |  | K_Sr_Ti | 13.81750956 |
|  |  |  | K_Sr_Zn | 13.81750956 |
|  |  |  | K_Ti_Zn | 13.81750956 |
|  |  |  | Mg_Mn_Re | 13.81750956 |
|  |  |  | Mg_Mn_Sn | 13.81750956 |
|  |  |  | Mg_Mo_Re | 13.81750956 |
|  |  |  | Mg_Mo_Sn | 13.81750956 |
|  |  |  | Mg_Mo_Sr | 13.81750956 |
|  |  |  | Mg_P_Ti | 13.81750956 |
|  |  |  | Mg_Re_S | 13.81750956 |
|  |  |  | Mg_Re_Sb | 13.81750956 |
|  |  |  | Mg_Re_Sn | 13.81750956 |
|  |  |  | Mg_Re_Ti | 13.81750956 |
|  |  |  | Mg_Re_Zn | 13.81750956 |
|  |  |  | Mg_S_Sr | 13.81750956 |
|  |  |  | Mg_Sb_Sn | 13.81750956 |
|  |  |  | Mg_Sn_Sr | 13.81750956 |
|  |  |  | Mg_Sn_Ti | 13.81750956 |
|  |  |  | Mg_Sr_Ti | 13.81750956 |
|  |  |  | Mg_Sr_Zn | 13.81750956 |
|  |  |  | Mg_Ti_Zn | 13.81750956 |
|  |  |  | Mn_Mo_Sr | 13.81750956 |
|  |  |  | Mn_Mo_Ti | 13.81750956 |
|  |  |  | Mn_Re_Ti | 13.81750956 |
|  |  |  | Mn_S_Sb | 13.81750956 |
|  |  |  | Mn_S_Sn | 13.81750956 |
|  |  |  | Mn_S_Ti | 13.81750956 |
|  |  |  | Mn_Sb_Ti | 13.81750956 |
|  |  |  | Mn_Se_Ti | 13.81750956 |
|  |  |  | Mn_Sn_Ti | 13.81750956 |
|  |  |  | Mn_Sr_Ti | 13.81750956 |
|  |  |  | Mn_Ti_Zn | 13.81750956 |
|  |  |  | Mo_P_Re | 13.81750956 |
|  |  |  | Mo_P_Ti | 13.81750956 |
|  |  |  | Mo_Re_Zn | 13.81750956 |
|  |  |  | Mo_S_Sb | 13.81750956 |
|  |  |  | Mo_S_Sn | 13.81750956 |
|  |  |  | Mo_Sb_Zn | 13.81750956 |
|  |  |  | Mo_Se_Sn | 13.81750956 |
|  |  |  | Mo_Se_Ti | 13.81750956 |
|  |  |  | Mo_Sn_Sr | 13.81750956 |
|  |  |  | Mo_Sn_Zn | 13.81750956 |
|  |  |  | Mo_Sr_Ti | 13.81750956 |
|  |  |  | Mo_Ti_Zn | 13.81750956 |
|  |  |  | P_S_Ti | 13.81750956 |
|  |  |  | P_Se_Sr | 13.81750956 |
|  |  |  | Re_S_Se | 13.81750956 |
|  |  |  | Re_S_Sn | 13.81750956 |
|  |  |  | Re_S_Ti | 13.81750956 |
|  |  |  | Re_Sb_Se | 13.81750956 |
|  |  |  | Re_Se_Sn | 13.81750956 |
|  |  |  | Re_Sn_Ti | 13.81750956 |
|  |  |  | S_Sb_Sn | 13.81750956 |
|  |  |  | S_Se_Sn | 13.81750956 |
|  |  |  | S_Se_Sr | 13.81750956 |
|  |  |  | S_Sr_Ti | 13.81750956 |
|  |  |  | S_Ti_Zn | 13.81750956 |
|  |  |  | Sb_Sn_Sr | 13.81750956 |
|  |  |  | Sb_Sr_Ti | 13.81750956 |
|  |  |  | Sb_Sr_Zn | 13.81750956 |
|  |  |  | Sb_Ti_Zn | 13.81750956 |
|  |  |  | Se_Sn_Sr | 13.81750956 |
|  |  |  | Se_Sn_Ti | 13.81750956 |
|  |  |  | Se_Sr_Zn | 13.81750956 |
|  |  |  | Sn_Sr_Ti | 13.81750956 |
|  |  |  | Sn_Ti_Zn | 13.81750956 |

| **The highly connected two ion modules in type 2 diabetes** | |  | **The highly connected three ion modules in type 2 diabetes** | |
| --- | --- | --- | --- | --- |
| **Ion module** | **score** |  | **Ion module** | **score** |
| Cu_P | 221.0801529 |  | Cr_Cu_P | 138.1750956 |
| Cr_P | 165.8101147 |  | Cu_P_Sn | 96.7225669 |
| Cr_Cu | 151.9926051 |  | Cu_Mo_P | 82.90505734 |
| Cu_Sn | 138.1750956 |  | Cu_P_Sb | 82.90505734 |
| P_Sn | 138.1750956 |  | Cu_P_Sr | 82.90505734 |
| Mn_P | 124.357586 |  | Mg_Mo_P | 82.90505734 |
| Mo_P | 124.357586 |  | Cr_Mo_P | 69.08754778 |
| Fe_Sr | 110.5400765 |  | Mg_P_S | 69.08754778 |
| Mg_P | 110.5400765 |  | Mg_S_Sn | 69.08754778 |
| P_Re | 110.5400765 |  | Mg_S_Zn | 69.08754778 |
| Cu_Mo | 96.7225669 |  | P_Re_Sn | 69.08754778 |
| Cu_Sr | 96.7225669 |  | Cr_Cu_Mn | 55.27003823 |
| Fe_P | 96.7225669 |  | Cr_Cu_Mo | 55.27003823 |
| Fe_Zn | 96.7225669 |  | Cr_Cu_Sb | 55.27003823 |
| Mg_S | 96.7225669 |  | Cr_Cu_Sr | 55.27003823 |
| P_S | 96.7225669 |  | Cr_Mg_P | 55.27003823 |
| P_Sb | 96.7225669 |  | Cr_Mn_P | 55.27003823 |
| P_Sr | 96.7225669 |  | Cr_P_S | 55.27003823 |
| P_Zn | 96.7225669 |  | Cr_P_Sb | 55.27003823 |
| S_Sn | 96.7225669 |  | Cr_P_Sr | 55.27003823 |
| S_Zn | 96.7225669 |  | Cr_P_Zn | 55.27003823 |
| Cr_S | 82.90505734 |  | Cu_Fe_P | 55.27003823 |
| Cu_Mg | 82.90505734 |  | Cu_Mg_P | 55.27003823 |
| Cu_Mn | 82.90505734 |  | Cu_Mg_S | 55.27003823 |
| Cu_S | 82.90505734 |  | Cu_Mg_Zn | 55.27003823 |
| Cu_Sb | 82.90505734 |  | Cu_Mn_P | 55.27003823 |
| Cu_Zn | 82.90505734 |  | Cu_P_Re | 55.27003823 |
| Fe_Mo | 82.90505734 |  | Cu_P_S | 55.27003823 |
| Mg_Mo | 82.90505734 |  | Cu_P_Zn | 55.27003823 |
| Mg_Zn | 82.90505734 |  | Cu_Re_Sn | 55.27003823 |
| Mn_Re | 82.90505734 |  | Cu_S_Sn | 55.27003823 |
| Mn_Sn | 82.90505734 |  | Mg_Mo_S | 55.27003823 |
| P_Se | 82.90505734 |  | Mg_Mo_Zn | 55.27003823 |
| Sb_Sr | 82.90505734 |  | Mg_P_Zn | 55.27003823 |
| Cr_Mn | 69.08754778 |  | Mn_P_Re | 55.27003823 |
| Cr_Mo | 69.08754778 |  | Mn_P_Sn | 55.27003823 |
| Cr_Sb | 69.08754778 |  | Mo_P_S | 55.27003823 |
| Cr_Zn | 69.08754778 |  | Mo_P_Zn | 55.27003823 |
| Cu_Fe | 69.08754778 |  | Mo_S_Zn | 55.27003823 |
| Cu_Re | 69.08754778 |  | P_S_Sn | 55.27003823 |
| Fe_Re | 69.08754778 |  | P_Sb_Sr | 55.27003823 |
| Fe_S | 69.08754778 |  | P_Se_Sn | 55.27003823 |
| Fe_Sb | 69.08754778 |  | Ca_Cu_Zn | 41.45252867 |
| Fe_Sn | 69.08754778 |  | Cr_Cu_Mg | 41.45252867 |
| Mg_Sn | 69.08754778 |  | Cr_Cu_Re | 41.45252867 |
| Mn_Sr | 69.08754778 |  | Cr_Cu_S | 41.45252867 |
| Mo_S | 69.08754778 |  | Cr_Cu_Sn | 41.45252867 |
| Mo_Sr | 69.08754778 |  | Cr_Cu_Zn | 41.45252867 |
| Mo_Zn | 69.08754778 |  | Cr_Mg_Mo | 41.45252867 |
| Re_Sn | 69.08754778 |  | Cr_Mg_S | 41.45252867 |
| Se_Sn | 69.08754778 |  | Cr_Mo_S | 41.45252867 |
| Sn_Zn | 69.08754778 |  | Cr_P_Re | 41.45252867 |
| Cr_Mg | 55.27003823 |  | Cr_P_Sn | 41.45252867 |
| Cr_Re | 55.27003823 |  | Cr_S_Zn | 41.45252867 |
| Cr_Sn | 55.27003823 |  | Cu_Fe_Sb | 41.45252867 |
| Cr_Sr | 55.27003823 |  | Cu_Fe_Sr | 41.45252867 |
| Fe_Mn | 55.27003823 |  | Cu_Mg_Mo | 41.45252867 |
| Fe_Se | 55.27003823 |  | Cu_Mg_Sn | 41.45252867 |
| Mo_Re | 55.27003823 |  | Cu_Mn_Re | 41.45252867 |
| Mo_Sb | 55.27003823 |  | Cu_Mn_Sn | 41.45252867 |
| Mo_Sn | 55.27003823 |  | Cu_P_Se | 41.45252867 |
| Re_Zn | 55.27003823 |  | Cu_S_Zn | 41.45252867 |
| Sb_Sn | 55.27003823 |  | Cu_Sb_Sr | 41.45252867 |
| Sn_Sr | 55.27003823 |  | Cu_Sb_Zn | 41.45252867 |
| Ca_Cu | 41.45252867 |  | Cu_Se_Sn | 41.45252867 |
| Ca_Sb | 41.45252867 |  | Cu_Sn_Sr | 41.45252867 |
| Ca_Sn | 41.45252867 |  | Cu_Sn_Zn | 41.45252867 |
| Ca_Zn | 41.45252867 |  | Fe_Mn_Re | 41.45252867 |
| Cr_Fe | 41.45252867 |  | Fe_Mo_Sb | 41.45252867 |
| Cu_Se | 41.45252867 |  | Fe_Mo_Zn | 41.45252867 |
| Fe_Ti | 41.45252867 |  | Fe_P_S | 41.45252867 |
| Mg_Mn | 41.45252867 |  | Fe_P_Sb | 41.45252867 |
| Mg_Re | 41.45252867 |  | Fe_P_Se | 41.45252867 |
| Mg_Sb | 41.45252867 |  | Fe_P_Sn | 41.45252867 |
| Mn_Zn | 41.45252867 |  | Fe_P_Sr | 41.45252867 |
| P_Ti | 41.45252867 |  | Fe_P_Zn | 41.45252867 |
| Re_Sr | 41.45252867 |  | Fe_S_Zn | 41.45252867 |
| S_Sb | 41.45252867 |  | Fe_Sb_Sr | 41.45252867 |
| Sb_Se | 41.45252867 |  | Fe_Se_Sn | 41.45252867 |
| Sb_Zn | 41.45252867 |  | Fe_Sn_Sr | 41.45252867 |
| Se_Ti | 41.45252867 |  | Mg_Mo_Re | 41.45252867 |
| Sr_Zn | 41.45252867 |  | Mg_P_Re | 41.45252867 |
| Ca_Mg | 27.63501911 |  | Mg_P_Sb | 41.45252867 |
| Ca_Mn | 27.63501911 |  | Mg_P_Sn | 41.45252867 |
| Ca_S | 27.63501911 |  | Mg_Sn_Zn | 41.45252867 |
| Ca_Sr | 27.63501911 |  | Mo_P_Re | 41.45252867 |
| Cr_K | 27.63501911 |  | P_Re_Zn | 41.45252867 |
| Cr_Se | 27.63501911 |  | P_S_Zn | 41.45252867 |
| Cu_K | 27.63501911 |  | P_Sb_Zn | 41.45252867 |
| Fe_Mg | 27.63501911 |  | P_Se_Ti | 41.45252867 |
| K_Mo | 27.63501911 |  | S_Sn_Zn | 41.45252867 |
| K_P | 27.63501911 |  | Sb_Se_Sn | 41.45252867 |
| K_Sr | 27.63501911 |  | Ca_Cu_Mg | 27.63501911 |
| Mn_Mo | 27.63501911 |  | Ca_Cu_S | 27.63501911 |
| Mn_S | 27.63501911 |  | Ca_Cu_Sn | 27.63501911 |
| Mn_Sb | 27.63501911 |  | Ca_Mg_S | 27.63501911 |
| Mn_Se | 27.63501911 |  | Ca_Mg_Sn | 27.63501911 |
| Re_S | 27.63501911 |  | Ca_Mg_Zn | 27.63501911 |
| Re_Sb | 27.63501911 |  | Ca_S_Sn | 27.63501911 |
| S_Sr | 27.63501911 |  | Ca_S_Zn | 27.63501911 |
| Se_Sr | 27.63501911 |  | Ca_Sb_Sr | 27.63501911 |
| Se_Zn | 27.63501911 |  | Ca_Sn_Zn | 27.63501911 |
| Sr_Ti | 27.63501911 |  | Cr_Cu_Fe | 27.63501911 |
| Ca_Cr | 13.81750956 |  | Cr_Cu_K | 27.63501911 |
| Ca_Fe | 13.81750956 |  | Cr_Fe_S | 27.63501911 |
| Ca_K | 13.81750956 |  | Cr_K_P | 27.63501911 |
| Ca_Mo | 13.81750956 |  | Cr_K_Sr | 27.63501911 |
| Ca_P | 13.81750956 |  | Cr_Mg_Zn | 27.63501911 |
| Ca_Se | 13.81750956 |  | Cr_Mn_Re | 27.63501911 |
| Cr_Ti | 13.81750956 |  | Cr_Mo_Zn | 27.63501911 |
| Cu_Ti | 13.81750956 |  | Cr_P_Se | 27.63501911 |
| Fe_K | 13.81750956 |  | Cr_Re_Sn | 27.63501911 |
| K_Mn | 13.81750956 |  | Cr_Re_Zn | 27.63501911 |
| K_Sb | 13.81750956 |  | Cr_S_Sb | 27.63501911 |
| K_Se | 13.81750956 |  | Cr_S_Sn | 27.63501911 |
| K_Sn | 13.81750956 |  | Cr_Sb_Sn | 27.63501911 |
| Mg_Sr | 13.81750956 |  | Cr_Sb_Sr | 27.63501911 |
| Mn_Ti | 13.81750956 |  | Cr_Sb_Zn | 27.63501911 |
| Mo_Se | 13.81750956 |  | Cu_Fe_S | 27.63501911 |
| Mo_Ti | 13.81750956 |  | Cu_Fe_Se | 27.63501911 |
| Re_Se | 13.81750956 |  | Cu_Fe_Sn | 27.63501911 |
| S_Se | 13.81750956 |  | Cu_K_P | 27.63501911 |
| Sb_Ti | 13.81750956 |  | Cu_K_Sr | 27.63501911 |
| Sn_Ti | 13.81750956 |  | Cu_Mg_Sb | 27.63501911 |
| Ti_Zn | 13.81750956 |  | Cu_Mo_S | 27.63501911 |
|  |  |  | Cu_Mo_Sn | 27.63501911 |
|  |  |  | Cu_Mo_Sr | 27.63501911 |
|  |  |  | Cu_Mo_Zn | 27.63501911 |
|  |  |  | Cu_S_Sb | 27.63501911 |
|  |  |  | Cu_S_Sr | 27.63501911 |
|  |  |  | Cu_Sb_Se | 27.63501911 |
|  |  |  | Cu_Sb_Sn | 27.63501911 |
|  |  |  | Cu_Se_Sr | 27.63501911 |
|  |  |  | Fe_Mg_Mo | 27.63501911 |
|  |  |  | Fe_Mg_P | 27.63501911 |
|  |  |  | Fe_Mg_Zn | 27.63501911 |
|  |  |  | Fe_Mn_Sr | 27.63501911 |
|  |  |  | Fe_Mn_Zn | 27.63501911 |
|  |  |  | Fe_Mo_P | 27.63501911 |
|  |  |  | Fe_Mo_Re | 27.63501911 |
|  |  |  | Fe_Mo_S | 27.63501911 |
|  |  |  | Fe_Mo_Sn | 27.63501911 |
|  |  |  | Fe_Mo_Sr | 27.63501911 |
|  |  |  | Fe_P_Re | 27.63501911 |
|  |  |  | Fe_P_Ti | 27.63501911 |
|  |  |  | Fe_Re_Sr | 27.63501911 |
|  |  |  | Fe_Re_Zn | 27.63501911 |
|  |  |  | Fe_S_Sb | 27.63501911 |
|  |  |  | Fe_S_Sn | 27.63501911 |
|  |  |  | Fe_S_Sr | 27.63501911 |
|  |  |  | Fe_Sb_Se | 27.63501911 |
|  |  |  | Fe_Sb_Sn | 27.63501911 |
|  |  |  | Fe_Se_Sr | 27.63501911 |
|  |  |  | Fe_Se_Ti | 27.63501911 |
|  |  |  | Fe_Sn_Zn | 27.63501911 |
|  |  |  | Fe_Sr_Ti | 27.63501911 |
|  |  |  | Fe_Sr_Zn | 27.63501911 |
|  |  |  | K_P_Sr | 27.63501911 |
|  |  |  | Mg_Mn_P | 27.63501911 |
|  |  |  | Mg_Mn_S | 27.63501911 |
|  |  |  | Mg_Mn_Sn | 27.63501911 |
|  |  |  | Mg_Mo_Sb | 27.63501911 |
|  |  |  | Mg_Mo_Sn | 27.63501911 |
|  |  |  | Mg_Re_S | 27.63501911 |
|  |  |  | Mg_Re_Zn | 27.63501911 |
|  |  |  | Mn_Mo_P | 27.63501911 |
|  |  |  | Mn_P_Se | 27.63501911 |
|  |  |  | Mn_P_Sr | 27.63501911 |
|  |  |  | Mn_Re_Sn | 27.63501911 |
|  |  |  | Mn_Re_Sr | 27.63501911 |
|  |  |  | Mn_S_Sn | 27.63501911 |
|  |  |  | Mn_Sb_Sr | 27.63501911 |
|  |  |  | Mn_Sn_Zn | 27.63501911 |
|  |  |  | Mn_Sr_Zn | 27.63501911 |
|  |  |  | Mo_P_Sb | 27.63501911 |
|  |  |  | Mo_P_Sn | 27.63501911 |
|  |  |  | Mo_P_Sr | 27.63501911 |
|  |  |  | Mo_Re_S | 27.63501911 |
|  |  |  | Mo_Re_Sr | 27.63501911 |
|  |  |  | Mo_Re_Zn | 27.63501911 |
|  |  |  | Mo_S_Sn | 27.63501911 |
|  |  |  | Mo_Sb_Sr | 27.63501911 |
|  |  |  | P_Re_S | 27.63501911 |
|  |  |  | P_Re_Sb | 27.63501911 |
|  |  |  | P_S_Sb | 27.63501911 |
|  |  |  | P_S_Sr | 27.63501911 |
|  |  |  | P_Sb_Se | 27.63501911 |
|  |  |  | P_Sb_Sn | 27.63501911 |
|  |  |  | P_Se_Sr | 27.63501911 |
|  |  |  | P_Se_Zn | 27.63501911 |
|  |  |  | P_Sn_Sr | 27.63501911 |
|  |  |  | P_Sn_Zn | 27.63501911 |
|  |  |  | Re_S_Zn | 27.63501911 |
|  |  |  | Re_Sn_Zn | 27.63501911 |
|  |  |  | S_Sb_Sn | 27.63501911 |
|  |  |  | S_Sb_Sr | 27.63501911 |
|  |  |  | Se_Sn_Sr | 27.63501911 |
|  |  |  | Ca_Cr_Cu | 13.81750956 |
|  |  |  | Ca_Cr_P | 13.81750956 |
|  |  |  | Ca_Cr_Sb | 13.81750956 |
|  |  |  | Ca_Cr_Sr | 13.81750956 |
|  |  |  | Ca_Cr_Zn | 13.81750956 |
|  |  |  | Ca_Cu_Mn | 13.81750956 |
|  |  |  | Ca_Cu_P | 13.81750956 |
|  |  |  | Ca_Cu_Sb | 13.81750956 |
|  |  |  | Ca_Cu_Sr | 13.81750956 |
|  |  |  | Ca_Fe_K | 13.81750956 |
|  |  |  | Ca_Fe_Mo | 13.81750956 |
|  |  |  | Ca_Fe_Sb | 13.81750956 |
|  |  |  | Ca_Fe_Se | 13.81750956 |
|  |  |  | Ca_Fe_Sn | 13.81750956 |
|  |  |  | Ca_K_Mo | 13.81750956 |
|  |  |  | Ca_K_Sb | 13.81750956 |
|  |  |  | Ca_K_Se | 13.81750956 |
|  |  |  | Ca_K_Sn | 13.81750956 |
|  |  |  | Ca_Mg_Mn | 13.81750956 |
|  |  |  | Ca_Mn_S | 13.81750956 |
|  |  |  | Ca_Mn_Sb | 13.81750956 |
|  |  |  | Ca_Mn_Sn | 13.81750956 |
|  |  |  | Ca_Mn_Sr | 13.81750956 |
|  |  |  | Ca_Mn_Zn | 13.81750956 |
|  |  |  | Ca_Mo_Sb | 13.81750956 |
|  |  |  | Ca_Mo_Se | 13.81750956 |
|  |  |  | Ca_Mo_Sn | 13.81750956 |
|  |  |  | Ca_P_Sb | 13.81750956 |
|  |  |  | Ca_P_Sr | 13.81750956 |
|  |  |  | Ca_P_Zn | 13.81750956 |
|  |  |  | Ca_Sb_Se | 13.81750956 |
|  |  |  | Ca_Sb_Sn | 13.81750956 |
|  |  |  | Ca_Sb_Zn | 13.81750956 |
|  |  |  | Ca_Se_Sn | 13.81750956 |
|  |  |  | Ca_Sr_Zn | 13.81750956 |
|  |  |  | Cr_Cu_Se | 13.81750956 |
|  |  |  | Cr_Fe_Mn | 13.81750956 |
|  |  |  | Cr_Fe_P | 13.81750956 |
|  |  |  | Cr_Fe_Re | 13.81750956 |
|  |  |  | Cr_Fe_Sb | 13.81750956 |
|  |  |  | Cr_Fe_Sr | 13.81750956 |
|  |  |  | Cr_Fe_Zn | 13.81750956 |
|  |  |  | Cr_K_Mn | 13.81750956 |
|  |  |  | Cr_K_Mo | 13.81750956 |
|  |  |  | Cr_Mg_Re | 13.81750956 |
|  |  |  | Cr_Mg_Sb | 13.81750956 |
|  |  |  | Cr_Mg_Sn | 13.81750956 |
|  |  |  | Cr_Mn_Mo | 13.81750956 |
|  |  |  | Cr_Mn_Se | 13.81750956 |
|  |  |  | Cr_Mn_Sn | 13.81750956 |
|  |  |  | Cr_Mn_Sr | 13.81750956 |
|  |  |  | Cr_Mn_Ti | 13.81750956 |
|  |  |  | Cr_Mo_Re | 13.81750956 |
|  |  |  | Cr_Mo_Sn | 13.81750956 |
|  |  |  | Cr_Mo_Sr | 13.81750956 |
|  |  |  | Cr_P_Ti | 13.81750956 |
|  |  |  | Cr_Re_S | 13.81750956 |
|  |  |  | Cr_Re_Sb | 13.81750956 |
|  |  |  | Cr_Re_Se | 13.81750956 |
|  |  |  | Cr_S_Sr | 13.81750956 |
|  |  |  | Cr_Sb_Se | 13.81750956 |
|  |  |  | Cr_Se_Sn | 13.81750956 |
|  |  |  | Cr_Se_Ti | 13.81750956 |
|  |  |  | Cr_Se_Zn | 13.81750956 |
|  |  |  | Cr_Sn_Zn | 13.81750956 |
|  |  |  | Cr_Sr_Zn | 13.81750956 |
|  |  |  | Cu_Fe_Mg | 13.81750956 |
|  |  |  | Cu_Fe_Mn | 13.81750956 |
|  |  |  | Cu_Fe_Mo | 13.81750956 |
|  |  |  | Cu_Fe_Re | 13.81750956 |
|  |  |  | Cu_Fe_Ti | 13.81750956 |
|  |  |  | Cu_Fe_Zn | 13.81750956 |
|  |  |  | Cu_K_Mn | 13.81750956 |
|  |  |  | Cu_K_Mo | 13.81750956 |
|  |  |  | Cu_Mg_Mn | 13.81750956 |
|  |  |  | Cu_Mn_Mo | 13.81750956 |
|  |  |  | Cu_Mn_S | 13.81750956 |
|  |  |  | Cu_Mn_Sr | 13.81750956 |
|  |  |  | Cu_Mn_Zn | 13.81750956 |
|  |  |  | Cu_Mo_Sb | 13.81750956 |
|  |  |  | Cu_P_Ti | 13.81750956 |
|  |  |  | Cu_Re_Sb | 13.81750956 |
|  |  |  | Cu_Re_Se | 13.81750956 |
|  |  |  | Cu_Re_Zn | 13.81750956 |
|  |  |  | Cu_S_Se | 13.81750956 |
|  |  |  | Cu_Se_Ti | 13.81750956 |
|  |  |  | Cu_Se_Zn | 13.81750956 |
|  |  |  | Cu_Sn_Ti | 13.81750956 |
|  |  |  | Cu_Sr_Ti | 13.81750956 |
|  |  |  | Cu_Sr_Zn | 13.81750956 |
|  |  |  | Fe_K_Mo | 13.81750956 |
|  |  |  | Fe_K_Sb | 13.81750956 |
|  |  |  | Fe_K_Se | 13.81750956 |
|  |  |  | Fe_K_Sn | 13.81750956 |
|  |  |  | Fe_Mg_Re | 13.81750956 |
|  |  |  | Fe_Mg_S | 13.81750956 |
|  |  |  | Fe_Mg_Sb | 13.81750956 |
|  |  |  | Fe_Mg_Sn | 13.81750956 |
|  |  |  | Fe_Mn_P | 13.81750956 |
|  |  |  | Fe_Mn_Sn | 13.81750956 |
|  |  |  | Fe_Mo_Se | 13.81750956 |
|  |  |  | Fe_Mo_Ti | 13.81750956 |
|  |  |  | Fe_Re_S | 13.81750956 |
|  |  |  | Fe_Re_Sn | 13.81750956 |
|  |  |  | Fe_S_Se | 13.81750956 |
|  |  |  | Fe_Sb_Ti | 13.81750956 |
|  |  |  | Fe_Sb_Zn | 13.81750956 |
|  |  |  | Fe_Se_Zn | 13.81750956 |
|  |  |  | Fe_Sn_Ti | 13.81750956 |
|  |  |  | Fe_Ti_Zn | 13.81750956 |
|  |  |  | K_Mn_P | 13.81750956 |
|  |  |  | K_Mn_Sr | 13.81750956 |
|  |  |  | K_Mo_P | 13.81750956 |
|  |  |  | K_Mo_Sb | 13.81750956 |
|  |  |  | K_Mo_Se | 13.81750956 |
|  |  |  | K_Mo_Sn | 13.81750956 |
|  |  |  | K_Mo_Sr | 13.81750956 |
|  |  |  | K_Sb_Se | 13.81750956 |
|  |  |  | K_Sb_Sn | 13.81750956 |
|  |  |  | K_Se_Sn | 13.81750956 |
|  |  |  | Mg_Mn_Mo | 13.81750956 |
|  |  |  | Mg_Mn_Re | 13.81750956 |
|  |  |  | Mg_Mn_Sb | 13.81750956 |
|  |  |  | Mg_Mn_Sr | 13.81750956 |
|  |  |  | Mg_Mn_Zn | 13.81750956 |
|  |  |  | Mg_Mo_Sr | 13.81750956 |
|  |  |  | Mg_P_Sr | 13.81750956 |
|  |  |  | Mg_Re_Sb | 13.81750956 |
|  |  |  | Mg_Re_Sn | 13.81750956 |
|  |  |  | Mg_Re_Sr | 13.81750956 |
|  |  |  | Mg_Sb_Sr | 13.81750956 |
|  |  |  | Mg_Sb_Zn | 13.81750956 |
|  |  |  | Mn_Mo_Re | 13.81750956 |
|  |  |  | Mn_Mo_Sb | 13.81750956 |
|  |  |  | Mn_Mo_Sr | 13.81750956 |
|  |  |  | Mn_P_S | 13.81750956 |
|  |  |  | Mn_P_Sb | 13.81750956 |
|  |  |  | Mn_P_Ti | 13.81750956 |
|  |  |  | Mn_Re_Sb | 13.81750956 |
|  |  |  | Mn_Re_Zn | 13.81750956 |
|  |  |  | Mn_S_Zn | 13.81750956 |
|  |  |  | Mn_Se_Sn | 13.81750956 |
|  |  |  | Mn_Se_Ti | 13.81750956 |
|  |  |  | Mn_Sn_Sr | 13.81750956 |
|  |  |  | Mo_Re_Sb | 13.81750956 |
|  |  |  | Mo_Re_Sn | 13.81750956 |
|  |  |  | Mo_Sb_Se | 13.81750956 |
|  |  |  | Mo_Sb_Sn | 13.81750956 |
|  |  |  | Mo_Sb_Ti | 13.81750956 |
|  |  |  | Mo_Sb_Zn | 13.81750956 |
|  |  |  | Mo_Se_Sn | 13.81750956 |
|  |  |  | Mo_Sn_Sr | 13.81750956 |
|  |  |  | Mo_Sn_Zn | 13.81750956 |
|  |  |  | Mo_Sr_Ti | 13.81750956 |
|  |  |  | P_Re_Se | 13.81750956 |
|  |  |  | P_Re_Sr | 13.81750956 |
|  |  |  | P_S_Se | 13.81750956 |
|  |  |  | P_Sn_Ti | 13.81750956 |
|  |  |  | P_Sr_Ti | 13.81750956 |
|  |  |  | P_Sr_Zn | 13.81750956 |
|  |  |  | P_Ti_Zn | 13.81750956 |
|  |  |  | Re_S_Sn | 13.81750956 |
|  |  |  | Re_Sb_Se | 13.81750956 |
|  |  |  | Re_Sb_Sn | 13.81750956 |
|  |  |  | Re_Sb_Sr | 13.81750956 |
|  |  |  | Re_Sb_Zn | 13.81750956 |
|  |  |  | Re_Se_Sn | 13.81750956 |
|  |  |  | Re_Se_Zn | 13.81750956 |
|  |  |  | Re_Sr_Zn | 13.81750956 |
|  |  |  | S_Sb_Se | 13.81750956 |
|  |  |  | S_Se_Sn | 13.81750956 |
|  |  |  | S_Se_Sr | 13.81750956 |
|  |  |  | S_Sn_Sr | 13.81750956 |
|  |  |  | Sb_Se_Sr | 13.81750956 |
|  |  |  | Sb_Se_Zn | 13.81750956 |
|  |  |  | Sb_Sn_Sr | 13.81750956 |
|  |  |  | Sb_Sn_Zn | 13.81750956 |
|  |  |  | Sb_Sr_Ti | 13.81750956 |
|  |  |  | Sb_Sr_Zn | 13.81750956 |
|  |  |  | Se_Sn_Ti | 13.81750956 |
|  |  |  | Se_Sn_Zn | 13.81750956 |
|  |  |  | Se_Sr_Ti | 13.81750956 |
|  |  |  | Se_Ti_Zn | 13.81750956 |
|  |  |  | Sn_Sr_Ti | 13.81750956 |
|  |  |  | Sn_Sr_Zn | 13.81750956 |
